# A Phase II Trial of Sorafenib in Metastatic Melanoma with Tissue Correlates

**DOI:** 10.1371/journal.pone.0015588

**Published:** 2010-12-29

**Authors:** Patrick A. Ott, Anne Hamilton, Christina Min, Sara Safarzadeh-Amiri, Lauren Goldberg, Joanne Yoon, Herman Yee, Michael Buckley, Paul J. Christos, John J. Wright, David Polsky, Iman Osman, Leonard Liebes, Anna C. Pavlick

**Affiliations:** 1 Department of Medical Oncology, New York University School of Medicine, New York, New York, United States of America; 2 Department of Pathology, New York University School of Medicine, New York, New York, United States of America; 3 Department of Dermatology, New York University School of Medicine, New York, New York, United States of America; 4 Sydney Cancer Centre, Royal Prince Alfred Hospital, Sydney, Australia; 5 Sydney Melanoma Unit and University of Sydney, Sydney, Australia; 6 Division of Biostatistics and Epidemiology, Weill Cornell Medical College, New York, New York, United States of America; 7 Investigational Drug Branch, National Cancer Institute, Bethesda, Maryland, United States of America; Universidade de São Paulo, Brazil

## Abstract

**Background:**

Sorafenib monotherapy in patients with metastatic melanoma was explored in this multi-institutional phase II study. In correlative studies the impact of sorafenib on cyclin D1 and Ki67 was assessed.

**Methodology/Principal Findings:**

Thirty-six patients treatment-naïve advanced melanoma patients received sorafenib 400 mg p.o. twice daily continuously. Tumor BRAF^V600E^ mutational status was determined by routine DNA sequencing and mutation-specific PCR (MSPCR). Immunohistochemistry (IHC) staining for cyclin D1 and Ki67 was performed on available pre- and post treatment tumor samples. The main toxicities included diarrhea, alopecia, rash, mucositis, nausea, hand-foot syndrome, and intestinal perforation. One patient had a RECIST partial response (PR) lasting 175 days. Three patients experienced stable disease (SD) with a mean duration of 37 weeks. Routine BRAF^V600E^ sequencing yielded 27 wild-type (wt) and 6 mutant tumors, whereas MSPCR identified 12 wt and 18 mutant tumors. No correlation was seen between BRAF^V600E^ mutational status and clinical activity. No significant changes in expression of cyclin D1 or Ki67 with sorafenib treatment were demonstrable in the 15 patients with pre-and post-treatment tumor samples.

**Conclusions/Significance:**

Sorafenib monotherapy has limited activity in advanced melanoma patients. BRAF^V600E^ mutational status of the tumor was not associated with clinical activity and no significant effect of sorafenib on cyclin D1 or Ki67 was seen, suggesting that sorafenib is not an effective BRAF inhibitor or that additional signaling pathways are equally important in the patients who benefit from sorafenib.

**Trial registration:**

Clinical Trials.gov NCT00119249

## Introduction

Metastatic melanoma is an aggressive skin cancer that is largely resistant to most systemic treatments. Response rates (RR) with dacarbazine, which remains the reference standard agent for advanced disease, have been in the 8–12% range in recent large, randomized trials [1], [2], [3]. Consequently, alternative treatments are being intensively investigated. Promising alternative approaches include strategies to target specific oncogenes and oncogenic pathways.

Activating mutations of the BRAF oncogene have been found in 50–60% of primary melanoma, metastatic melanoma tissues, and melanoma cell lines by us and other groups [4], [5], [6]. BRAF^V600E^, which accounts for more than 90% of BRAF mutations, leads to constitutive activation of downstream signaling via the MAPK cascade, a pathway that is critical in cell cycle regulation and proliferation [7]. Inhibition of the activated MAPK pathway through BRAF silencing with RNA interference results in apoptosis of melanoma cell lines carrying the BRAF^V600E^ mutation [8], [9], [10] and regression of BRAF^V600E^ melanoma xenografts [11], suggesting that BRAF is an attractive drug target.

Sorafenib is an orally active multikinase agent that inhibits BRAF and CRAF as well as a number of other cellular targets such as vascular endothelial growth factor receptors (VEGFR, particularly VEGFR-2), platelet-derived growth factor receptor (PDGFR), FMS-like tyrosine kinase 3 (Flt-3); c-Kit protein (c-Kit); and RET receptor tyrosine kinases [12], [13]. It has recently gained FDA approval for use in advanced renal cell carcinoma [14] and hepatocellular carcinoma [15]. In preclinical studies, sorafenib was shown to block the activation of the MAPK downstream molecules pMEK and pERK and to slow tumor growth in human melanoma xenograft models [9], [16]. No PR's were seen in 37 advanced melanoma patients who were treated with sorafenib monotherapy as part of a randomized discontinuation trial [17]. In a randomized phase II trial, the combination of dacarbazine and sorafenib demonstrated superior objective responses and progression-free survival (but no improved overall survival) [18], whereas a phase III trial of the Eastern Oncology Group (ECOG), E2603, randomizing advanced melanoma patients to paclitaxel and carboplatin with or without sorafenib recently failed its primary endpoint of overall survival in a planned interim analysis [19].

The primary rationale of the current study was to explore the safety and efficacy of sorafenib monotherapy in patients with metastatic melanoma. Correlative studies were conducted to investigate the effect of this treatment on cyclin D1 or Ki67. We also aimed to assess an association between BRAF^V600E^ mutational status in metastatic melanoma patients and response to sorafenib therapy.

## Methods

The protocol for this trial and supporting CONSORT checklist are available as supporting information; see Checklist S1 and Protocol S1.

### Ethics

This study was conducted according to the principles expressed in the Declaration of Helsinki. The protocol (NCI study number NCI-6617, Clinical Trials.gov identifier NCT00119249) received prior approval by the institutional review board at New York University Langone Medical Center. The protocol was reviewed by the local institutional review board at each participating institution, and all patients provided written informed consent.

### Participants

Patients were eligible if they were at least 18 years of age, had histologically or cytologically confirmed, unresectable, stage III or stage IV melanoma and a life expectancy ≥3 months. All patients had measurable disease according to the international criteria proposed by the Response Evaluation Criteria in Solid Tumors (RECIST) committee [20]. Other eligibility criteria were adequate performance status (Eastern Cooperative Oncology Group 0–2 or Karnofsky ≥60%), adequate renal, hepatic, and bone marrow function (serum creatinine ≤1.5 times the upper limit of normal [ULN]), total bilirubin ≤2 times ULN, AST (SGOT) and ALT (SGPT) ≤2.5 times ULN, absolute neutrophil count ≥1.5×10^9^/l, platelet count ≥100×10^9^/l). Patients with brain metastases were eligible if they were steroid-independent with radiographically stable lesions for at least 6 weeks after whole brain radiation and no mass effect present radiographically at the time of study entry. Patients who had received previous chemotherapy for metastatic disease were not enrolled. Pregnant or nursing patients and patients who were receiving any other investigational agents were excluded from the study. Exclusion criteria also comprised a history of serious allergic reactions to eggs (sorafenib is formulated using egg phospholipids), disorders that would interfere with oral intake of the study drug, HIV positive patients receiving combination anti-retroviral therapy (due to possible pharmacokinetic interactions with the study drug), patients with an active infection or with other indications of poor medical risk, and patients with any evidence of bleeding diatheses. The study was conducted by the New York Cancer Consortium (www.newyorkcancerconsortium.org). The participating institutions were New York University Langone Medical Center, New York, NY and Sydney Cancer Center, Sydney, Australia.

### Interventions

All patients received sorafenib orally at a dose of 400 mg twice a day on days 1 to 28 (one treatment cycle). Treatment cycles were repeated every 28 days until unacceptable toxicity, disease progression, or death. Growth factor support was used as per American Society of Clinical Oncology guidelines. Sorafenib was dose-reduced by 200 mg per day if patients had clinically significant hematologic or other adverse events as measured by revised NCI Common Toxicity Criteria (CTC) version 3.0 and the events were felt to be attributable to sorafenib. More than 2 dose reductions or dose re-escalation was not allowed; treatment was discontinued if any grade 3 or 4 toxicity did not resolve within 3 weeks.

### Objectives

The objectives of this single arm, phase II trial were to assess the efficacy and toxicity of sorafenib in metastatic melanoma patients. Furthermore, the impact of sorafenib on cyclin D1 and Ki67 as well as BRAF^V600E^ mutational status was to be assessed. The hypotheses were that sorafenib has efficacy in this patient population that cyclin D1 and Ki67 are impacted in melanoma tissue, and that clinical responses are associated with the presence of a BRAF^V600E^ mutation.

### Outcomes

The primary endpoint was RR, while secondary endpoints were time to progression (TTP) and toxicity. Prior to each treatment cycle, patients were evaluated by medical history, physical examination, assessment of Eastern Cooperative Oncology Group performance status, complete blood counts, and serum chemistries. Tumor response was assessed after every 2 cycles (8 weeks) of treatment using appropriate imaging (computer-assisted tomography (CT), positron-emission-tomography combined with CT, or magnetic resonance imaging) and outcomes were evaluated according to RECIST criteria. Stable disease had to last at least 6 months in order to be qualified as such [21]. Duration of a partial response (PR) was defined as the time that the response was first documented until progression, whereas duration of stable disease (SD) was defined as the time from treatment initiation until progression. Patients were treated until disease progression or development of unacceptable toxicities.

Attempts were made to obtain punch biopsies of soft tissue melanoma lesions from all patients on day 1 (pre-treatment) and day 28 of the first cycle. Immunohistochemistry staining for cyclin D1 and Ki67 was performed on paraffin embedded tissue from day 1 and day 28 biopsy samples. BRAF^V600E^ mutation analysis (as described below) and H&E staining for confirmation of the melanoma diagnosis was performed on paraffin embedded tissue from day 1 punch biopsy specimens in all patients.

### Correlative Studies

Patient tissue was analyzed for the presence of BRAF^V600E^ mutation with both conventional DNA sequencing and the more sensitive mutation-specific PCR (MSPCR) as described previously [6], [22]. Briefly, DNA was extracted from paraffin embedded tumor tissue using QIAamp Mini DNA kit (QIAGEN). The melanoma cell line SK-MEL29 [23] was used as BRAF^V600E^ positive control, human placental DNA (Sigma-Aldrich, St. Louis, MO) as negative control. The forward, mutant-specific primer has 2 bases at the 3′ end that do not anneal to the wild-type sequence, whereas the mutant sequence allows annealing of the terminal 3′ base, resulting in specific amplification of the V600E mutant allele. The reverse primer was labeled with HEX fluorochrome to allow detection using an ABI 310 Genetic Analyzer (Applied Biosystems). For conventional sequencing, the entire BRAF exon 15 was amplified using primers as previously described [4]. Exon 11 was not amplified.

Immunohistochemistry (IHC) was performed on formalin fixed, paraffin embedded 3 micron tissue sections using mouse anti-cyclin D1 (clone P2D11F11) and mouse anti-Ki-67 (clone K-2) (Ventana Medical Systems Tucson, AZ USA). In brief, sections were deparaffinized in xylene (3 changes), rehydrated through graded alcohols (3 changes 100% ethanol, 3 changes 95% ethanol) and rinsed in distilled water. Heat induced epitope retrieval was performed in 10mM citrate buffer pH 6.0 in a 1200-Watt microwave oven at 90% power. All antibodies were retrieved for 20 minutes and sections were allowed to cool for 30 minutes followed by rinsing in distilled water. Antibody incubations and detection were carried out at 37°C on a NEXes instrument (Ventana Medical Systems Tucson, Arizona) using Ventana's reagent buffer and detection kits unless otherwise noted. Endogenous peroxidase activity was blocked with hydrogen peroxide. Cyclin D1 and Ki67 were pre-diluted and incubated for 30 minutes. Primary antibodies were detected with Ventana's biotinylated goat anti-mouse or goat anti-rabbit secondary antibody, respectively. After secondary antibody application, streptavidin-horseradish-peroxidase conjugate was applied. The complex was visualized with 3,3 diaminobenzidene and enhanced with copper sulfate. Slides were washed in distilled water, counterstained with hematoxylin, dehydrated and mounted with permanent media. Appropriate positive and negative controls were included with the study sections.

For assessment of IHC staining, five representative tumor sections (pre-treatment day 1 and post treatment day 28) per tissue specimen were selected by a board-certified pathologist (using H&E and IHC-stained samples). The individual cells positive for cyclin D1 and Ki67, and total number of tumor cells were counted manually at 40× magnification using photographs taken from the individual tumor sections selected by the pathologist. As part of a variability assessment, one investigator counted all samples and a second investigator re-counted one of the 5 (randomly chosen) replicate tumor sections per specimen. Inter-observer variability for these confirmatory counts was measured as the difference between two readings in percent of the mean. The ratios of the marker positive cells/total tumor cells were calculated from averages of the five different tumor sections.

### Sample Size and Statistical Design

In each subgroup defined by BRAF^V600E^ status, a Simon two-stage minimax design was initially proposed to test the null hypothesis that response rate at the end of the second cycle (56 days) was less than or equal to 5% versus the alternative hypothesis that the response rate was greater than or equal to 20%. If the drug was not effective, there was a 3.3% probability of concluding that it was (type I alpha error). If the drug was effective, there was a 14.2% probability of concluding that it was not (type II beta error). After testing the drug on 13 patients per BRAF subgroup in the first stage, the plan was to terminate the BRAF stratum (subgroup) if 0 patients per subgroup responded. If the trial proceeded to the 2^nd^ stage (for each subgroup), then 37 patients per BRAF subgroup were to be evaluated for response. If the total number responding was less than or equal to 4 per subgroup, the drug was to be rejected for the relevant subgroup. The overall response rate along with subgroup-specific response rates were to be estimated at the end of the trial along with 95% confidence intervals. However, due to accrual limitations, the two-stage design was applied to the single cohort of 36 patients accrued (i.e., comparing 5% versus 20% response rates using the same alpha and beta errors as indicated above).

Secondary endpoints of progression-free survival (i.e., time to progression) and toxicity were analyzed for the cohort. Progression-free survival was calculated using the Kaplan-Meier method and a 95% confidence interval (95% CI) was estimated for the median progression-free survival time. Patients with available pre-treatment and post-treatment correlative markers (cyclin D1 and Ki67) were compared by the Wilcoxon signed-rank test. All analyses were performed in SAS version 9.2 (SAS Institute, Inc., Cary, NC) and Stata version 10.0 (Stata Corporation, College Station, TX).

## Results

### Recruitment

Between August 2005 and September 2007, 36 patients were enrolled at 2 centers in the United States and Autralia. Patients were followed until disease progression or discontinuation of treatment due to unacceptable side effects, intercurrent illness, or patient withdrawal.

### Baseline Data

The baseline clinical characteristics for the study population are summarized in table 1. Median age was 64 years (range, 22 to 91 years); all patients but one had an ECOG performance status of 0–1. Patients with cutaneous melanoma (31), mucosal melanoma (3), ocular melanoma (1), and melanoma of unknown primary (1) were enrolled. Seventeen patients had LDH values 1.1× normal or higher. Staging was performed according to the American Joint Committee on Cancer guidelines. Thirty four patients had stage IV disease and 2 patients had unresectable stage IIIC disease.

**Table 1:** Patient demographics and disease characteristics.

Parameter	No.		%
*Sex*			
Male	20		56
Female	16		44
*Age, years*			
Median		64	
Range		22–91	
*Race*			
Caucasian	35		97
Asian	1		3
*Primary site*			
cutaneous	32		89
mucosal	3		8
ocular	1		3
*Stage III*			
IIIc	2		6
*Stage IV*			
M1a	11		31
M1b	6		17
M1c	17		47
*Metastatic site No*			
1	7		19
2	15		42
3 or more	14		39
*Site of metastasis*			
Lung	18		50
Liver	8		22
Bone	6		17
Skin	3		8
Soft tissue	20		56
Lymph nodes (distant)	24		67
Other	8		17
*LDH*			
within normal limits	19		53
elevated	17		47
*ECOG Performance Status*			
0	28		78
1	7		19
2	1		3

### Numbers analyzed

All of the 36 patients who were enrolled were analyzed for toxicity and clinical response (intention to treat). One out of the 36 patients developed rapidly progressive disease prior to initiation of therapy and never received treatment with sorafenib.

### Outcomes and estimation

**Figure 1:**
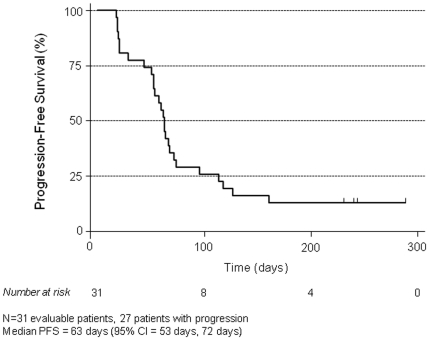
Kaplan-Meier analysis of progression-free survival.

The median duration of treatment was 63 days. Complete responses (CR) were not seen in any of the 36 patients; one patient had a PR that lasted 175 days. Three patients had SD that lasted between 239 and 288 days (mean 233 days). The response rate (the primary endpoint of the study) was 1/36 = 2.8% (95% CI = 0.07%, 14.5%). The disease control rate (CR+PR+SD) was 4/36 = 11.1% (95% CI = 3.1%, 26.1%). Median TTP for assessable patients was 63 days (95% CI = 53 days, 72 days) (Fig. 1). All patients with either PR or SD who were assessable for response received treatment until the time of documented disease progression. Five patients could not be assessed for response: Three patients withdrew consent prior (2) or shortly after (1) initiation of treatment, one patient had rapid disease progression within 2 days of study enrollment, and one patient had a severe adverse event related to sorafenib prior to the first scheduled response assessment.

### Correlative studies

BRAF mutational analysis was performed using both conventional DNA sequencing and MSPCR. Six out of 33 patients (18%) for whom BRAF analysis was available tested positive for the BRAF^V600E^ mutation by sequencing, whereas 18/30 (60%) patients were found to carry the mutation as measured by MSPCR. The cutaneous melanoma patient who experienced a PR had a BRAF^V600E^ mutation as tested by both methods (Table 3). The low disease control rate precludes conclusions about a correlation between mutational status and response to sorafenib therapy. All 3 patients with SD were BRAF wild-type by both methods (Table 3). Individual patients with information on tumor characteristics, BRAF^V600E^ status, stage and extension of disease, and tumor response are listed in table 4.

**Figure 2:**
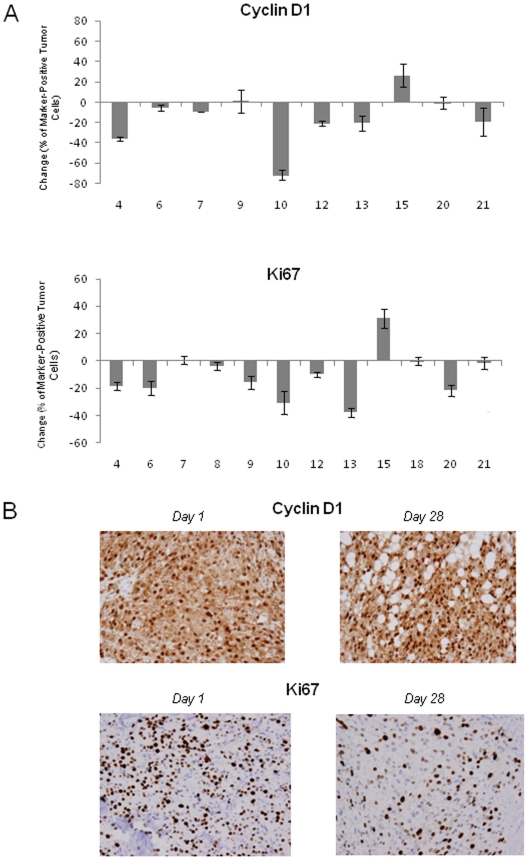
Cyclin D1 and Ki67 expression in tumor biopsies obtained before and after treatment with sorafenib. **A**: Increase/decrease in the percentage of tumor cells staining for cyclin D1 and Ki67 between pre-treatment (day 1) and post-treatment (day 28) samples, as measured by immunohistochemistry. The numbers represent absolute changes in percentage of cells. **B**: Representative stainings for cyclin D1 and Ki67 from patient #20 are shown (40×).

Immunohistochemistry staining for cyclin D1 and Ki67 was performed on a subset of patients on day 1 (pre-treatment) and day 28 of the first treatment cycle. We counted 3848±2264 (mean ± standard deviation) tumor cells per patient sample (763±463 on average per replicate). Inter-observer variability was <6% on average for the random replicate sample that was counted by a second investigator. Pre- and post-treatment samples were available for 15 patients, however in 3 of the post-treatment specimens, no tumor was seen. Expression of cyclin D1 and Ki67 was not significantly changed with sorafenib treatment in patients from which paired pre- and post-treatment samples were available; the median % change was −14 for cyclin D1 (p = 0.18) and −13 for Ki67 (p = 0.06) (Fig. 2).

### Adverse Events

Sorafenib was well tolerated and most adverse events were grade 1 or 2, reversible, clinically easily manageable, and did not require dose reductions (Table 2). The most common adverse events (possibly, probably, or definitely related) were limited to the skin and gastrointestinal systems as listed in table 2. Two patients discontinued sorafenib because of treatment-related adverse events (hand-foot syndrome and rash). One patient experienced grade II hypertension and one patient with metastatic disease to the small bowel experienced grade III intestinal perforation. There were no reported grade IV adverse events.

**Table 2:** Toxicity profile.

	Toxicity grade			
	Grade 1 and 2		Grade 3	
	n	%	n	%
*Constitutional*				
Fatigue	5	13	1	3
Anorexia	3	8	0	0
*Gastrointestinal*				
Mucositis	6	16	0	0
Diarrhea	11	30	0	0
Intestinal perforation	0	0	1	3
Nausea/Vomiting	6	16	0	0
*Dermatological*				
Hand-Foot syndrome	6	16	2	5
Rash	17	46	2	5
Alopecia	8	22	0	0
Flushing	3	8	0	0
Dry skin	6	16	0	0
*Neurological*				
Neuropathy	3	8	0	0
Pain	8	22	0	0
*Miscellaneous*				
Dyspnea	0	0	1	3
Hypertension	2	5	0	0

**Table 3:** Tumor responses and BRAF mutational status.

Response	BRAF^V600E^			
	by sequencing		by MSPCR	
	wt	mutant	wt	mutant
**PR**	0	1	0	1
**SD**	3	0	3	0
**PD**	22	3	8	16

**Table 4:** Tumor characteristics and response to sorafenib for individual patients.

#	Age	Gender	BRAF^V600E^ (sequ)	BRAF^V600E^ (MSPCR)	Primary site	metastatic site	# of sites	AJCC Stage	LDH	Response
1	79	F	W	M	cutaneous	ST, liver, bone, lymph node	4	IVM1c	nl	PD
2	91	M	W	W	cutaneous	skin, lymph node	2	IVM1a	nl	NE
3	69	M	M	M	cutaneous	ST, lung	2	IVM1b	nl	PR
4	71	M	W	M	cutaneous	lymph node	1	IVM1a	nl	PD
5	22	M	W	M	cutaneous	ST, liver, lung, kidney, spine	5	IVM1c	h	NE
6	67	F	W	M	cutaneous	ST, lung, liver, lymph node	4	IVM1c	h	PD
7	53	m	W	W	ocular	ST, lymph node, liver, lung	4	IVM1a	nl	PD
8	49	M	W	M	cutaneous	ST, lymph node	2	IVM1a	nl	PD
9	47	F	M	M	cutaneous	ST, lung, bone, adrenal, lymph node	5	IVM1c	h	PD
10	84	M	W	W	cutaneous	ST, lymph node	2	IVM1c	h	PD
11	42	M	W	W	cutaneous	skin, lung	2	IVM1b	h	SD
12	62	M	W	W	cutaneous	ST, lung, liver, kidney, gall bladder	5	IVM1c	h	SD
13	59	F	W	W	cutaneous	ST, lymph node	2	IVM1a	nl	SD
14	59	F	W	W	mucosal	ST, lymph node	2	IVM1c	h	PD
15	61	F	W	W	cutaneous	skin	1	IVM1a	nl	PD
16	77	M	W	W	cutaneous	liver, lung, lymph node	3	IVM1c	h	PD
17	55	F	M	na	unknown	lung, lymph node	2	IVM1c	h	NE
18	79	M	W	M	cutaneous	ST, bone, lymph node	3	IVM1c	h	PD
19	68	M	W	W	mucosal	liver, lung, adrenal, lymph node	4	IVM1c	h	PD
20	42	F	W	M	cutaneous	lung, bone, skin, ST, lymph node	5	IVM1c	nl	PD
21	75	M	W	W	cutaneous	lymph node	1	IIIc	h	PD
22	68	M	W	M	cutaneous	liver, adrenal, ST, lymph node	4	IVM1c	h	PD
23	72	M	W	M	cutaneous	lung, adrenal, mediastinum, ST, brain	5	IVM1c	h	PD
24	47	M	na	na	cutaneous	lung	1	IVM1b	nl	NE
25	65	F	W	W	cutaneous	soft tissue	1	IVM1a	nl	PD
26	83	M	W	M	cutaneous	soft tissue, lymph node	2	IVM1a	nl	PD
27	71	M	W	M	cutaneous	soft tissue	1	IVM1c	h	PD
28	71	F	M	M	cutaneous	lymph node, soft tissue, lung	3	IVM1c	h	PD
29	37	M	W	M	cutaneous	soft tissue, lung	2	IVM1b	nl	PD
30	34	F	M	na	cutaneous	lymph node, liver	2	IVM1a	nl	NE
31	75	M	W	na	cutaneous	lymph node, ST, spleen, kidney	4	IVM1c	h	PD
32	34	F	M	M	cutaneous	soft tissue, bone	2	IIIc	nl	PD
33	57	F	na	na	cutaneous	soft tissue, skin	2	IVM1a	nl	PD
34	53	F	w	M	cutaneous	soft tissue, lung	2	IVM1b	nl	PD
35	64	F	na	na	cutaneous	lymph node, lung	2	IVM1b	nl	PD
36	54	F	W	M	mucosal	vulva, cervix	2	IVM1a	nl	PD

MSPCR: mutant specific PCR; h∶high; na: not available; AJCC: American Joint Committee on Cancer.

## Discussion

The primary objective of this trial was the evaluation of efficacy and tolerability of sorafenib monotherapy in patients with advanced melanoma. The disease control rate (CR+PR+SD) of 11.1% (4/36) suggests that sorafenib has no meaningful activity as a single agent in metastatic melanoma patients (the primary endpoint of the study was response rate). With respect to previously described activity of sorafenib in melanoma, monotherapy lead to tumor growth inhibition, but not tumor shrinkage in melanoma tumor xenografts [9], [16]. Furthermore, no PR's were seen in 37 advanced melanoma patients who were treated with sorafenib monotherapy as part of a randomized discontinuation trial [17]. The toxicity profile was similar to that noted in other clinical trials using sorafenib.

The importance of MAPK pathway upregulation and the high prevalence of the BRAF^V600E^ mutation in melanoma patients in conjunction with preclinical data showing that BRAF blockade results in apoptosis and tumor control in preclinical models provided the rationale for investigating sorafenib in this trial. The most obvious explanation for the limited activity of sorafenib in melanoma as a single agent is its lack of specificity and relatively low effectiveness as a BRAF kinase inhibitor. New compounds with higher selectivity and potency for mutant BRAF, PLX4720 [24] and PLX4032 [10], are in preclinical and clinical development. PLX4032 has been studied as single agent in patients with solid tumors (mainly melanoma patients) and responses, including complete responses, were seen in the majority of patients whose tumors were harboring the BRAF^V600E^ mutation [25]. If an upregulated MAPK pathway is in fact the most important mechanism that drives tumor proliferation in melanomas carrying the BRAF^V600E^ mutation, the high selectivity and potency of these new agents should provide more insight into the role of BRAF as a critical therapeutic target in melanoma.

An explanation for the (albeit low) activity of sorafenib independent of BRAF inhibition might be explained by the effect of sorafenib on other targets, such as VEGFR2 (which has recently been associated with response to sorafenib given in combination with chemotherapy in metastatic melanoma patients [26]), or c-KIT. Of note, there were 2 patients with an unconfirmed PR who had a mucosal melanoma. One of these 2 patients (subject #19) tested negative for c-kit mutations in exons 11, 13, 17, and 18, while no information on the c-kit status of subject #14 is available. Approximately 40% of patients with mucosal melanoma have c-kit aberrations in exons 11, 13, and 17, and clinical responses, including CR's, were seen in these patients after treatment with imatinib [27], [28], [29], [30]. Notably, a recent case report documented a PR in a patient with the D820Y mutation in exon 17 of *KIT* who was treated with sorafenib [31]. Pharmacogenomic studies to elucidate the signature of targets inhibited by sorafenib in tumor tissues are underway as part of ongoing clinical trials in melanoma and other tumor types.

Noteworthy is a recent report by Karreth et al., showing that CRAF inhibits BRAF^V600E^ kinase activity and that sorafenib at low doses, through its inhibitory effect on CRAF, can lead to increased MAPK pathway activation [32]. These findings were recently corroborated and expanded by 2 groups, reporting that ATP competitive RAF inhibition, through transactivation of the non-inhibited member of CRAF-CRAF homodimers or CRAF-BRAF heterodimers, leads to increased signaling through the RAF-MEK-ERK pathway in BRAF wild-type tumors, resulting in increased tumor growth [33], [34]. This evidence highlights the importance of knowledge of the BRAF status in patients treated with specific RAF inhibitors such as PLX4032. We speculate that these new findings could explain some of our findings in the study, such as upregulation of cyclin D1 and Ki67 after treatment with sorafenib in 2 patients and the absence of a correlation between BRAF status and clinical benefit in our patient population.

Sixty percent of patients had a melanoma carrying the BRAF^V600E^ mutation as assessed by MSPCR, which is comparable to the mutation rate previously seen in melanoma [4], [35], [36]. One explanation for the relatively low rate of the BRAF^V600E^ mutation by sequencing in our study could be contamination of the soft tissue metastases (from which all biopsies were obtained) with normal tissue. In a previous study, we showed that the sensitivity of DNA sequencing is much lower compared to MSPCR [22]. Of note, in that analysis soft tissue melanoma metastases had a lower BRAF^V600E^ mutation rate compared to lymph node and visceral metastases (32% vs. 45–50%). In our opinion a highly sensitive technique such as MSPCR or TaqMan Real Time PCR, using sequence specific probes (as is being used in the ongoing PLX4032 phase 3 trial [37]) is desirable and the preferred method over conventional DNA sequencing for BRAF mutation testing.

Expression of cyclin D1 and Ki67 (all proteins located downstream of the BRAF kinase) were analyzed by immunohistochemistry in paired tumor biopsies (pre- and post treatment) from 15 of 36 patients. Expression of the 2 markers was highly variable, ranging from 0–90% of tumor cells. Similar variability in expression levels of 10 other direct targets or downstream mediators of sorafenib was seen in a study of melanoma patients who were treated with sorafenib in addition to carboplatin and paclitaxel [19], [26]. When we compared pre- and post treatment tumor samples, the expression of the 2 markers was lower in the majority of post-treatment specimens (Fig. 2), however this did not reach statistical significance. The biologic relevance (whether this reflects a treatment effect) is not clear as the differences were low in absolute numbers mainly because of the low baseline expression in many of the tumors. It is important to emphasize that we did perform quantitative analysis on 5 different, full tumor sections for each tumor specimen, thus evaluating the staining of several thousand tumor cells for each sample. We are therefore confident that the numbers are accurate even in cases were low numbers of tumor cells staining for the respective markers were seen. The data suggest that sorafenib does not inhibit the MAPK pathway in the advanced melanoma patients studied here. Due to the low RR in this study we are unable to conclude whether tumor response was associated with a more pronounced decrease of cyclin D1 and Ki67 compared to non-responders.

It is well known that other pathways in addition to the MAPK cascade, such as the PI3K/AKT pathway, play a role in oncogenesis in melanoma, and inhibition of multiple pathways was shown to be synergistic in melanoma cell lines [38]. A number of these pathways are not affected by sorafenib and it has been proposed that rational combination of specific targeted agents depending on genetic subtypes of melanoma (for example BRAF^V600E^ mutant melanoma with other genetic aberrations such as PTEN deletion or AKT amplification) might be a promising strategy to tailor treatment for optimal efficacy in melanoma.

In conclusion, sorafenib at the evaluated dose of 400 mg twice daily has limited activity in metastatic melanoma as a single agent. BRAF^V600E^ mutational status was detected more frequently by MSPCR compared to conventional PCR sequencing. We did not find a significant impact on the MAPK pathway in the tumors as measured by immunohistochemical analysis of cyclin D1 and Ki67 after treatment with sorafenib. No evidence for correlation between BRAF^V600E^ mutational status of the tumor and clinical activity was found, suggesting that sorafenib is not an effective BRAF inhibitor or that additional sorafenib targets may play a role in the few patients who benefit from the drug.

## Supporting Information

Checklist S1 CONSORT checklist.
(DOC)

Protocol S1 Sorafenib trial protocol.
(DOC)
