# Supplementary material for: A Phase II Trial of Sorafenib in Metastatic Melanoma with Tissue Correlates
Source: PLoS One. 2010 Dec 29;5(12):e15588. doi: 10.1371/journal.pone.0015588 (PMC3012061; doi:10.1371/journal.pone.0015588)
Supplement: Protocol S1 — Sorafenib trial protocol. (DOC) [file pone.0015588.s002.doc]

NYU 04-38

NCI Protocol #: 6617

Amendment 1: 11/3/05

Amendment 2: 1/17/06 Disapproved

Amendment 3: 2/6/06

Amendment 4: 3/3/06 Disapproved

Amendment 5: 4/14/06 Disapproved

Amendment 6: 4/27/06

Amendment 7: 9/12/06

A Phase II Study of BAY 43-9006 (NCS 724772) in UNRESECTABLE STAGE III AND stage IV

Melanoma (IND 69,896)

**Coordinating Center:** New York Presbyterian/Weill Medical College of Cornell University

**Protocol Chair:** **Anna Pavlick, DO**

#### NYU School of Medicine

Bellevue C&D Building, Room 556

462 First Ave

New York, NY 10016

USA

Tel: 1 (212) 263 6485

e-mail: anna.pavlick@med.nyu.edu

**Co-Investigators:** **Franco Muggia, MD**

NYU School of Medicine

Bellevue C&D Building, Room 556

462 First Ave

New York, NY 10016

USA

Tel: 1 (212) 263 6485

e-mail: franco.muggia@med.nyu.edu

**Participating Institutions:**

### Tsiporah Shore, MD

NY Presbyterian/Cornell Medical Center

520 East 70^th^ Street

New York, NY 10021

Tel: (212) 746-2646

##### Anne L. Hamilton, MBBS

Sydney Cancer Centre

Royal Prince Alfred Hospital

Gloucester House

Missenden Rd

Camperdown, NSW 2050

Australia

Tel: 61 (2) 9515 7680

**Dr. Jane Beith**

Senior Staff Specialist, Dept of Medical Oncology   Sydney Cancer Centre,
 Royal Prince Alfred Hospital - GH6  Missenden Rd, Camperdown,  NSW, Australia
Tel:  + 61 2 9515 8304 Fax:  + 61 2 9519 1546
email: [jane.beith@cs.nsw.gov.au](mailto:jane.beith@cs.nsw.gov.au)

**Correlative Studies:** **Iman Osman, MD**

**David Polsky, MD, PhD**

**Peter Brooks, PhD**

**Leonard Liebes, PhD**

NYU School of Medicine

462 First Avenue

Bellevue Building C & D

New York NY 10016 USA

Tel: 1 (212) 263-6485

**Statistician:** **Judith D. Goldberg, ScD**

**Vandana Mukhi, MA**

NYU School of Medicine

660 First Avenue

New York, NY 10010

Tel: 1 (212) 263-6485

**Study Coordinator:** **Carole Rubenstein**

#### NYU School of Medicine

Bellevue C&D Building, Room 556

462 First Ave

New York, NY 10016

USA

Tel: 1 (212) 263 6485

Fax: 1 (212) 263 8210

e-mail: [rubenc01@med.nyu.edu](mailto:rubenc01@med.nyu.edu)

**NCI-Supplied Agent**: Bay 43-9006 (NSC 724772 )

**SCHEMA**

Two subgroups of patients will be treated.

Patients with stage IV melanoma will be treated with the BRAF inhibitor, BAY 43-9006. Patients will be stratified based on whether or not their tumors contain a B-RAF mutation.

**Subgroup A: No prior therapy, B-RAF+**

These patients have had no prior therapy and their melanoma tumors have a *BRAF* mutation.

**Subgroup B: No prior therapy, B-RAF-**

These patients have had no prior therapy and their tumors do not have a *BRAF* mutation (wild BRAF status).

B-RAF status can be determined from real-time biopsies or paraffin-embedded metastatic tumor obtained previously. The first 13 patients in each subgroup must have melanoma metastases amenable to biopsy before and after therapy and must consent to having these biopsies done.

**. Since there has been a preponderance of wild type patients accrued to date, the protocol will allow enrollment to any patient with or without biopsy accessible metastatic disease in an attempt to expediciously complete the trial.

All patients will receive continuous Bay 43-9006 400mg po BID. Efficacy will be evaluated in terms of measurable anti-tumor effects and proportion of clinical responses. Correlation of response with presence of BRAF mutation in the melanoma tumor will be determined and the hypothesis that treatment with BAY 43-9006 can disrupt the MAPK pathway by down regulating downstream proteins such as phosphor-ERK, CDK4, and cyclin D1 will be explored.

**TABLE OF CONTENTS**

Page

**SCHEMA**  3

**1. OBJECTIVES**  6

**2. BACKGROUND**  6

2.1 **Malignant Melanoma Stage IV**  6

2.2 **B-RAF/BAY 43-9006** 8

2.3 **Rationale**  15

**3. PATIENT SELECTION**  15

3.1 **Eligibility Criteria** 15

3.2 **Exclusion Criteria**  16

3.3 **Inclusion of Women and Minorities**  17

**4. TREATMENT PLAN**  17

4.1 **Agent Administration**  17

4.2 **Supportive Care Guidelines**  18

4.3 **Duration of Therapy**  18

**5. EXPECTED ADVERSE EVENTS/DOSE MODIFICATIONS**  18

5.1 **Expected Adverse Events Associated with BAY 43-9006**  18

5.2 **Dosing Delays/Dose Modifications**  19

**6. AGENT FORMULATION AND PROCUREMENT**  20

**7. CORRELATIVE/SPECIAL STUDIES**  22

**8. STUDY CALENDAR**  25

**9. MEASUREMENT OF EFFECT**  26

9.1 **Definitions**  26

9.2 **Guidelines for Evaluation of Measurable Disease**  28

9.3 **Response Criteria**  28

9.4 **Confirmatory Measurement/Duration of Response**  29

9.5 **Progression-Free Survival**  30

9.6 **Response Review**  30

**10. REGULATORY AND REPORTING REQUIREMENTS** 30

10.1 **Patient Registration** 30

10.2 **Expedited Adverse Event Reporting** 30

10.3 **Data Reporting**  32

10.4 **CTEP Multicenter Guidelines**  32

10.5 **Clinical Trials Agreement (CTA)** ………………………………………………….33

**11. STATISTICAL CONSIDERATIONS**  34

**12. REFERENCES** 36

**APPENDICES**

##### APPENDIX A

Eligibility Criteria…..………………………………………………………………….......39

##### APPENDIX B

Performance Status Criteria …..40

##### APPENDIX C

Genetic and Pharmacodynamic Endpoints with Sample Collection……………………..….41

**APPENDIX D**

Tumor Immunohistochemistry………………………………………………………………43

##### APPENDIX E

DNA Extraction……………………………………………………..………………………..44

**APPENDIX F**

Monitoring of Collagen Cryptic Epitopes……………………………………………….……45

##### APPENDIX G

Sample Data Collection Forms...…………………………………………………………46

**APPENDIX H**

Medication Diary……………………………………………………………………………48

1. OBJECTIVES

1.1 To assess the efficacy of BAY 43-9006, (anti-tumor effects and proportion of clinical responses), in stage IV malignant melanoma patients. .

1.2 To assess the relationship between efficacy of BAY 43-9006 and the presence of mutant BRAF and wild-type BRAF within the melanoma tumors.

1.3 To further characterize the known toxicity profile of BAY 43-9006 at the recommended phase II dose.

1.4 To determine in the first 13 patients of each subgroup if treatment with BAY 43-9006 disrupts the *RAS/BRAF/MEK/MAPK* pathway by depleting intra-tumor stores of BRAF and/or downstream proteins such as CDK4, phospho-erk, MAPK and cyclin D1.

1.5 To assess the relationship between serum cryptic collagen epitopes and extent of tumor burden, invasion and metastasis and potential of serum collagen epitopes to serve as a possible surrogate marker to monitor the course of disease following therapeutic intervention in all patients enrolled in the study.

**2. BACKGROUND**

**2.1 Malignant Melanoma Stage IV**

Melanoma is an increasingly important health problem. The incidence of melanoma has risen at a rate of 4% per year over the last two decades and now approaches 30 per 100,000 in some populations (1). Surgery can be curative in Stage I, II, or III disease, but a large number of patients with deep primary lesions or nodal involvement will develop extensive recurrence or distant metastases (stage IV disease). No curative treatment exists for stage IV melanoma. Dacarbazine (DTIC) or DTIC-containing regimens are the most commonly used treatments for advanced disease.

## 2.1.1 Standard Therapy

In the first-line chemotherapy treatment of patients with stage IV disease, agents with reproducible activity against melanoma include DTIC, cisplatin, nitrosoureas, and vinca alkaloids. DTIC is the most active single agent with response rates ranging from about 10% to 20%, and median response durations of 4 to 6 months (2). Although several recent studies using a combination of DTIC and other agents have shown higher response rates, these combinations have not proven to be superior to single agent DTIC for the general population. Similarly, a Phase III study comparing temozolomide to DTIC showed no substantial improvement in survival or in other primary clinical endpoint (3).

A variety of combination chemotherapy regimens have produced response rates of 30% to 50% in single-institution Phase 2 trials. Two of the more active regimens were the 3-drug combination of cisplatin/vinblastine/DTIC (CVD) (4) and the 4-drug combination of cisplatin/DTIC/BCNU/tamoxifen (CDBT) (5). However, in a randomized multi-institutional trial comparing CVD to DTIC alone, the CVD arm was not significantly superior in response rate, response duration, or survival (6). In a recent update of this trial that encompassed approximately 150 patients, the CVD arm produced a 19% response rate compared to 14% for DTIC alone with no difference in either response duration or survival. A randomized, Phase 3 trial (EST 91-140) also demonstrated no significant survival benefit associated with CDBT relative to DTIC alone.

Several recent studies have indicated potential value for the addition of either interferon-α or tamoxifen to DTIC (7-9). The actual benefit of the addition of interferon and/or tamoxifen to DTIC in patients with advanced melanoma was tested by the Eastern Cooperative Oncology Group (ECOG) in a large-scale, four-arm, Phase 3 trial (EST 3690). The overall response rate was 18% (range 12% to 21% for the 4 arms), the median time to treatment failure was 2.6 months, and median survival was 9.1 months. There was no increase in objective response, increased duration of time to progression, or survival advantage attributable to the addition of interferon, tamoxifen, or both to DTIC. Based on this trial and the cumulative data from prior studies, there is no compelling evidence to support the addition of either interferon or tamoxifen to DTIC in this disease.

## 2.1.2 Immunotherapy

Interest in the use of biologic response modifiers has been fostered by a variety of clinical and laboratory observations suggesting that host immunologic mechanisms may occasionally influence the course of melanoma. During the past decade, two biologic agents, interferon-α (IFN-α) and IL-2, have shown reproducible single agent anti-tumor activity against advanced melanoma. Both IL-2 and IFN-α have produced response rates in the 15 to 20% range (10,11). High dose IL-2 therapy, administered by intravenous bolus either alone or in combination with LAK cells, has produced durable complete responses in approximately 5% of patients (11-14). However, the known adverse effects of IL-2 have precluded wide application due to the increased risk of treatment-related morbidity.

## 2.1.3 Biochemotherapy Combinations

A number of investigators have studied combinations of cytotoxic chemotherapy with IL-2 based immunotherapy. In general, the best results have been observed in studies that combined DTIC- and/or cisplatin-based chemotherapy with either high-dose IL-2 alone, or lower doses of IL-2 combined with IFN-α.

Ongoing Phase 2 and 3 trials include CVD ± IL-2/IFN administered in a sequential fashion (M.D. Anderson Cancer Center), cisplatin/DTIC ± IL-2/IFN (NCI Surgery Branch), and E3695, the intergroup (ECOG/SWOG/CALGB) study of CVD ± IL-2/IFN. While the results of these studies will be important for patients with good performance status, the toxicity of these regimens will limit their applicability to the general population.

New agents that are better tolerated and more active against melanoma than platinum and DTIC are clearly desperately required.

- 1. **B-RAF/BAY 43-9006**
  2. **STRUCTURE OF BAY43-9006**

**BACKGROUND**

Activation of the RAS oncogene signaling pathway is considered to be an important mechanism by which human cancer develops. Ras regulates several pathways which synergistically induce cellular transformation, including the *RAS/BRAF/MEK/MAPK* cascade and the rac and rho pathways (15,16). In particular, Ras activates the *RAS/BRAF/MEK/MAPK* pathway by first localizing Raf to the plasma membrane, where Raf initiates a mitogenic kinase cascade. Activated Raf phosphorylates and activates Mek which in turn phosphorylates and activates Erk. Activated Erk then translocates from the cytoplasm into the nucleus and modulates gene expression via the phosphorylation of transcription factors. Thus activation of Raf kinase, via activation of Ras, is thought to play an important role in carcinogenesis.

In particular, BRAF, a serine/threonine kinase, has been shown to be activated in a number of human tumor types including melanoma, ovarian, and papillary thyroid carcinomas (17,18,19,20,21,22). A survey of 43 cancer cell lines showed that all B-raf mutations resided in exons 11 or 15. Remarkably, 80% of these B-raf mutations represent a single nucleotide change of T-A at nucleotide 1796 resulting in a valine to glutamic acid change at residue 599 (V599E, exon 15) in the CR3 domain (ATP binding and substrate recognition) which in turn confers constitutive kinase activity (17,18).

***In Vitro* Activity**

The ability of BAY 43-9006 to inhibit a number of kinases was evaluated by Panlabs (Investigator’s Brochure, 2002). The *in vitro* profile of BAY 43-9006 is summarized below:

| Assay | BAY 43-9006(IC_50_) |
| --- | --- |
| *In vitro* biochemical assay:c-Raf kinaseB-Raf kinaseMutant V599E B-RafMEK-1 kinaseERK-1 kinase | 2-6 nM25 nM38 nM33% (10,000 nM)24% (10,000 nM) |
| B-raf ER/3T3 cells mechanistic assayERK phoshorylation in HCT 116 | 4.5 µM2.5 μM |
| Selectivity vs Insulin tyrosine kinase receptor in cellsEGFRHER2/neu | 26% at 20 µMinactiveinactive |
| HCT-116 proliferation assay | 2.3 µM |
| Soft agar assay(HCT-116/ MiaPaCa cells) | 7.1/6.2 µM |

*In vitro* kinase assays demonstrated that BAY 43-9006 is a potent inhibitor of wild-type and mutant (V599E) B-Raf and c-Raf Kinase isoforms invitro (Bayer communication). In addition, BAY 43-9006 did not inhibit human EGFR or Her2 kinases at 10 μM nor PKC-α, PKC-β, PKC-γ, and PKA (rat, rabbit and bovine sources) kinase activity *in vitro*. BAY 43-9006 demonstrated an IC_50_ of 780 nM against p59 (bovine) Fyn kinase (Src family of protein tyrosine kinases). In non-kinase targets, BAY 43-9006 had moderate potency against the adenosine A3, dopamine D1, and muscarinic M3 receptors with an IC_50_ of 1.6 μM, 2.0 μM and 3.1 μM, respectively. In summary, BAY 43-9006 showed ≥ 100-fold more selectivity for raf kinase than for other target proteins.

***In Vivo* Activity**

BAY 43-9006 has demonstrated *in vivo* anti-tumor efficacy as a single agent against a broad range of human tumor xenografts as summarized in the following table. The models evaluated include HCT-116 and DLD-1 colon tumor xenografts, MX-1 mammary tumor xenograft, NCI-H460 and A549 NSCLC xenografts, MiaPaCa-2 pancreatic tumor xenografts, and SK-OV-3 ovarian tumor xenografts. In this table, compound efficacy is expressed as percent tumor growth inhibition (TGI) and is calculated as ((1-(T/C)) *100, where T and C represent the mean tumor size in the Treated and Control groups respectively at the first measurement after the end of treatment.

*BAY 43-9006 Demonstrates Broad Spectrum Anti-Tumor*

*Efficacy in Preclinical Xenograft Models*

| Tumor Type | Model | Dose (mg/kg/dose free base equiv.)^a^ | Percent TGI  ((1-(T/C))*100) |
| --- | --- | --- | --- |
| Colon | HCT-116 | 10  30  100 | 45  64  68 |
|  | DLD-1 | 15  30  60 | 31  66  75 |
| NSCLC | NCI-H460 | 10  30 | 27  56 |
|  | A549 | 30  60 | 60  68 |
| Mammary | MX-1 | 30  60 | 51  67 |
| Pancreatic | Mia-PaCa-2 | 10  30  100 | 45  66  73 |
| Ovarian | SK-OV-3 | 10  30  100 | 58  64  81 |

^a^ Compound dosed as BAY 43-9006 or equivalent dose levels of it tosylate salt, BAY 54-9085.

The majority of the initial anti-tumor efficacy evaluations *in vivo* were conducted in the HCT116 colon tumor model since the tumorigenicity of this cell line was previously shown to be dependent on K-ras activation. Additional studies indicated that prolonged anti-tumor efficacy could be attained by extending the duration of treatment and that, in this model, BAY 43-9006 was able to arrest tumor growth even if therapy was initiated against a substantial tumor burden.

BAY 43-9006 also showed significant oral activity against two additional human tumor xenograft models that contain K-ras mutations: MiaPaCa-2 pancreatic carcinoma and H460 non small cell lung carcinoma. The anti-tumor efficacy of BAY 43-9006 was also evaluated against the human SKOV-3 ovarian tumor cell line that contains a wild-type RAS, but overexpresses both the EGF and Her2 growth factor receptors. These receptors also signal through the RAS/RAF/MEK pathway.

Taken together, this data suggests that BAY 43-9006 may be of therapeutic value not only in human tumors containing *ras* gene mutations, but also in tumors with other activating mutatations in Ras/Raf/Mek pathway.

The ability of BAY 43-9006 (or its tosylate salt, BAY 54-9085) to be combined with paclitaxel, irinotecan, gemcitabine, or cisplatin was evaluated in preclinical *in vivo* models. In these studies, the focus was to evaluate if the co-administration of BAY 43-9006 would adversely affect the tolerance or anti-tumor efficacy of the ‘standard of care’ agent. The general health of mice was monitored and mortality was recorded daily. Tumor dimensions and body weights were recorded twice a week starting with the first day of treatment. Treatments producing greater than 20% lethality and/or 20% net body weight loss were considered ‘toxic’. The results of these combinability analyses are summarized below.

*Combinability of Concurrent Treatment with BAY 43-9006 and*

*Clinically Established Agents*

|  | **Combination Agent** | Tumor Model | **Combinability**  **Y/N** |
| --- | --- | --- | --- |
|  | Paclitaxel | NCI-H460 NSCLC  MX-1 Mammary | Yes  Yes |
|  | Irinotecan | DLD-1 Colon | Yes |
|  | Gemcitabine | MiaPaCa-2 Pancreatic | Yes |
|  | Cisplatin | NCI-H23 NSCLC | Yes |

BAY 43-9006 can be safely combined with a variety of standard cytotoxic cancer chemotherapy agents, including paclitaxel, irinotecan, gemcitabine and cisplatin with no significant increase in the toxicity associated with those agents and without diminishing their anti-tumor efficacy in preclinical models.

**PHASE 1 CLINICAL EXPERIENCE**

Four company-sponsored phase 1 studies (2 single agent, 2 combination) are currently determining the MTD, DLT pharmacokinetics (PK), pharmacodynamics, and the recommended phase 2 dose of BAY 43-9006. Currently, BAY 43-9006 is being administered twice daily orally (PO, BID). with several dosing schedules: continuous dosing, 4 weeks on/1 week off, 3 weeks on/1 week off, and 1 week on/1 week off.

Preliminary results from the 4 company-sponsored phase 1 studies are summarized below (23). A total of 172 patients in the 4 studies (median age 55; 74 colorectal, 12 breast, 12 hepatocellular carcinoma, 11 ovary, 10 GI/other, 9 renal, 6 pancreas, 2 head and neck, and 5 melanoma; M:F 96:76; PS 0:1:2 69:82:19) received BAY 43-9006 at dose levels up to 800 mg b.i.d. In a preliminary analysis of 115 patients receiving BAY 43-9006 ≥ 200 mg b.i.d., 38 patients had stable disease (SD) for at least 12 weeks. The median duration of treatment was 9.6+ weeks, 28 patients remained in the study for > 6 months, 9 patients continued for > 1 year, and 10 patients are still ongoing. Furthermore, 2 confirmed partial responses were observed on the 400 mg continuous treatment (hepatocellular carcinoma) and 600 mg 21 days on/7 days off (renal cell carcinoma) regimens. Four patients (1 colorectal, 2 ovarian, 1 renal) achieved ≥ 20% decrease in tumor size. Toxicities have been mild to moderate and included reversible skin rash, hand-foot syndrome, diarrhea and fatigue. DLT was diarrhea (800 mg b.i.d.), skin toxicity (800 mg b.i.d. 7days on/7 days off; 600 mg b.i.d. on other schedules), nausea, and hypertension (800 mg b.i.d. 7days on/7 days off). With respect to PK, C_max_ and AUC increased with increasing doses and considerable interpatient variability was noted. Steady state plasma levels of BAY 43-9006 were reached by the seventh day of dosing. Preliminary data across all studies indicate that the terminal half-life ranged from 16-53 hours . There was evidence of inhibition of the Raf kinase pathway in patients treated at higher doses (not specified).

A phase 1 study is determining the MTD, DLT, PK, and pharmacodynamics in patients with advanced, refractory solid tumors (24). BAY 43-9006 was administered on a regimen of repeat cycles of 1 week on/1 week off using b.i.d. dosing. There were 19 evaluable patients (8 colorectal, 4 sarcoma, 2 pancreas, 1 renal cell carcinoma, 1 melanoma, 1 mesothelioma, 1 cervical, and 1 unknown primary) at 5 dose levels; 100 mg (3 pts), 200 mg (3 pts), 400 mg (4 pts), 600 mg (6 pts), and 800 mg (3 pts). Grade 3 and 4 adverse events of dyspnea, hypertension, hyponatremia, increased prothrombin time, and rash were reported. Rash was observed at all dose levels. Toxicities were rapidly reversible and no myelosuppression was seen. There were 5 drug-related DLTs (at 800 mg); 2 patients with grade 2 rash, 2 patients with grade 3 rash, and 1 patient with grade 3 hypertension. Five patients (cervical, renal, sarcoma, melanoma, and colon) had stable disease. The melanoma patient showed decreased ^18^[F]-fluorodeoxyglucose uptake on PET scan prior to progression of disease. The renal cell patient is still ongoing, with SD > 13 months. An increase in C_max_ and AUC up to 400 mg BID was observed with increasing dose with a terminal half-life ranging from 16-53 hours. The MTD was established at 600 mg.

In one phase I/II combination trial with gemcitabine (25), BAY 43-9006 was administered PO b.i.d. on a continuous schedule at three different dose levels, along with gemcitabine at 1000 mg/m^2^ weekly × 7 weeks followed by one rest week (for cycle 1), then BAY 43-9006 was administered PO b.i.d. with gemcitabine at 1000 mg/m^2^ weekly × 3 weeks every 4 weeks (from cycle 2). Thirteen patients have been entered into 3 cohorts: 100 mg (3 pts), 200 mg (3 pts), and 400 mg (7 pts). Hematological adverse events were non-dose limiting and consistent with expected gemcitabine toxicity. Mild cases of elevated transaminase, mucositis, nausea. and flu-like symptoms were reported. One patient (at 400 mg) experienced dose-limiting grade 3 fatigue. There was a trend of increasing skin toxicity with higher BAY 43-9006 dose levels, most of which occurred within the first treatment cycle. Seven out of thirteen patients developed skin toxicity, including 2/3 patients at the 200 mg dose level (one grade 1, one grade 2), and 5/7 patients at the 400 mg dose level (four grade 1-2, one grade 3 hand-foot syndrome at cycle 2). Preliminary pharmacokinetic analysis revealed no significant interactions between BAY 43-9006 and gemcitabine. Prolonged SDs were observed in several tumor types (tumor type and duration not given). The RPTD is BAY 43-9006 400 mg b.i.d. and gemcitabine 1000 mg/m^2^.

A second phase 1 combination trial administered BAY 43-9006 with carboplatin and paclitaxel (26). BAY 43-9006 was given PO b.i.d. from days 2 to 19 out of a 21 day cycle, and carboplatin (AUC=6) and paclitaxel (225mg/m^2^) were administered on day 1. Fifteen patients (melanoma (9 pts), lung (1pt), sarcoma (1pt), basal cell (1pt), colon (1pt), esophagus (1pt) and renal cell (1pt)) accrued to 3 dose levels (7 pts at 100 mg, 3 pts at 200 mg, and 1 at 400mg). One grade 3 rash at the 100mg dose level resulted in expanding this cohort. Three patients were treated at the 200mg dose level and one patient at 400mg without DLT. In the first treatment cycle, adverse events (grades 3 and 4) were limited to grade 4 neutropenia (2 pts) and grade 3 events of thrombocytopenia (2), anemia (1), rash (1), hypersensitivity (1), and fatigue (1). All but two patients developed a rash. Among nine patients with melanoma, three partial responses (2 pts at 100 mg and 1 pt at 200 mg) and 5 SDs (2 pts >2 months) were reported. In addition, minor responses were observed in one basal cell carcinoma (at 100 mg after cycle 2 with progression after cycle 4), one SD (at 100 mg for 6+ cycles) in sarcoma, and one SD in renal cell carcinoma (at 200 mg for 3 cycles). Preliminary PK data indicate that there are no significant pharmacokinetic interactions between BAY 43-9006 and paclitaxel/carboplatin.

A single agent trial done at the Royal Mardsen Hospital was presented at the 2004 ASCO meeting. This was a Phase II, single agent trial in patients with Stage IV, refractory melanoma. Patients were treated with BAY 43-9006 400mg po BID for 12 weeks. Responses were assessed at 12 weeks. Twenty patients were enrolled between 10/02 and 4/03. 15 patients had progressive disease before 12 weeks. 4 evaluable patients were reported with 1 PR and 3 SD. 5 patients developed Grade III skin toxicity and 2 patients developed hypertension requiring intervention. No hematolgic toxicity was noted and all toxicities were reversible.

**NYU PRELIMINARY STUDIES:**


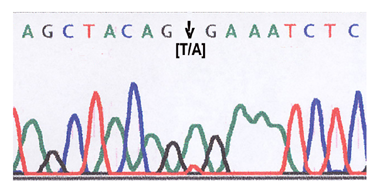


**Figure 1. BRAF HotSpot Mutation.** An electropherogram demonstrating the T1796A nucleotide substitution detected in a metastatic melanoma tissue sample.

We recently reported an analysis of *BRAF* and *N-RAS* mutations in 77 metastatic melanoma tumor specimens, and11 melanoma cell lines (27,28,29). In this study we amplified and sequenced *BRAF* exons 11 and 15, and *N-RAS* exons 2 and 3. We detected mutations in 36 of 77 (47%) tissue specimens and 8 of 11 (73%) cell lines. Ninety-two percent of mutations in tissues occurred in *BRAF* exon 15, and 31 of 33 (94%) exon 15 mutations consisted of the previously identified T1796A nucleotide substitution. An electropherogram demonstrating this mutation is shown in Figure1.

In studies preceding this application, we have developed a PCR amplification strategy that specifically amplifies the T1796A mutant. We have designed three different oligonucleotide primers specific for the mutation (mFwd, mRev1, mRev2). The primers are described in greater detail in the methods section of Aim #1. Briefly, these mutant-specific primers are used with intronic primers to amplify products that range in size from 151 bp to 172 bp. We have established the specificity of all three mutant-specific primers by testing them on melanoma cell lines SK-MEL 85 (wild-type for exon 15, mutant for exon 11), and SK-MEL 29 (mutant for exon 15). As shown in Figure 2, PCR reactions using each primer set were performed at annealing temperatures of 58^o^C, 60^o^C, and 63^o^C. Each primer showed specificity for the mutant product, as evidenced by disappearance of the wild-type band as the temperature increased.

Additional confirmation of the specificity of these primers will be obtained by analyzing the 9 other melanoma cell lines. As described in the methods section, we will develop this assay further with the use of fluorescently labeled intronic primers to further enhance sensitivity and specificity. We estimate that fluorescent detection of PCR products is approximately 200-fold more sensitive than detection using ethidium bromide-stained agarose gels. In Figure 3 serial dilutions of a fluorescently labeled PCR product were analyzed in an ethidium bromide-stained agarose gel and the ABI 310 Genetic analyzer. Quantitation of the PCR products in the agarose gel using a molecular mass ladder showed that reliable detection of the PCR products was far superior using the ABI 310 analyzer compared to the agarose gel.

58^o^C60^o^C 63^o^C

12345678 9M

400bp

200bp

100bp

261bp

168bp

261bp

172bp

**Figure 2. Multiplex PCR for mutant BRAF and p53 exon 4 (internal control).** DNA from SK-Mel 29 (BRAF mutant), lanes 2, 5, 8 or SK-Mel 85 (BRAF wild-type), lanes 3, 6, 9 was used as template to test the three mutant specific primers at 3 different annealing temperatures (Ta). Lanes 1-3, Ta=58^o^C; 4-6, Ta=60^o^C; 7-9, Ta=63^o^C. Each panel shows the result from a different primer. A. mRev2, B. mRev1, C. mFwd. In all three panels, the lower band is the product of the BRAF primers, the upper band, the p53 primers. Lanes 1, 4, and 7 are negative controls in which only the template DNA was omitted from the reaction.


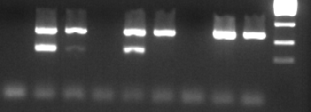

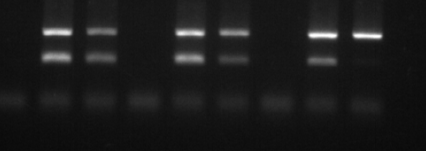

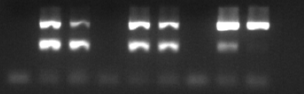


**A**

**B**

**C**

**2.3 Rationale**

The *RAS/BRAF/MEK/MAPK* pathway is one of the cell’s major pathways for transmitting extracellular growth factor signals to the nucleus. Signaling via this pathway upregulates Cyclin D**1, which binds to the CDK4/6 kinase, promoting phosphorylation of the retinoblastoma tumor suppressor protein (RB). Phosphorylation of RB releases it from members of the E2F family of transcription factors, allowing the activation of genes required for cells to progress from the G1 to S phase of the cell cycle.** Activating mutations in any of the kinases in the pathway would be expected to result in uncontrolled cell proliferation. *RAS* mutations are found at high frequency in some cancers (e.g. colon); however they are uncommon in most melanomas. The low frequency of *RAS* mutation in melanoma suggests that activation of the MAPK pathway may be accomplished by activating downstream elements. Our group has recently reported that activating *BRAF* mutations occurs in 48% of metastatic melanoma tissues (30). Other groups have reported frequencies of 21-67% of melanomas tested. All of the *BRA*F mutations occur in exons 11 or 15 which correspond to the G-loop and the activation segment of the kinase domain, respectively. Approximately 90% of *BRAF* mutations in melanoma are found at amino acid 599 (exon 15), constituting a mutational hotspot. The mutations produce a constitutively activated kinase, which, when transfected into fibroblasts, leads to cell transformation. In this trial, we propose to test an anti-*BRAF* drug BAY 43-9006. This drug directly inhibits RAF function; however, its specificity for this kinase is not yet established. Although the results of Phase I clinical trials have only been reported in abstract form, it appears that the drug can be administered safely using a weekly dosing schedule. To date, there is little information on whether this compound inhibits phosphorylated MAPK, and no data exist on the response of melanoma patients or other patients with tumors expressing activated *BRAF* mutation.

**3. PATIENT SELECTION**

**3.1 Eligibility Criteria (Appendix A)**

3.1.1 Patients must have histologically or cytologically confirmed unresectable Stage III and Stage IV melanoma. The first 13 patients in each subgroup must have melanoma tumors amenable to biopsy before and after therapy and they must consent to having these biopsies done. These biopsies are necessary in order to carry out the correlative pharmacodynamic measurements (see below). In most cases, biopsied tumors will be cutaneous or subcutaneous metastases. If additional patients are accrued beyond the first 13- patients in each subgroup, we will obtain either real time biopsies or paraffin embedded tissue from a previous biopsy for prospective identification of the B-RAF mutation. This is necessary in order to subgroup patients. Since there has been a preponderance of wild type patients accrued to date, the protocol will allow enrollment to any patient with or without biopsy accessible metastatic disease in an attempt to expediciously complete the trial.

3.1.2 Patients must have measurable disease, defined as at least one lesion that can be accurately measured in at least one dimension (longest diameter to be recorded) as >20 mm with conventional techniques or as >10 mm with spiral CT scan. See section 9.1.1 for the evaluation of measurable disease.

3.1.3 No prior systemic chemotherapy for metastatic disease is permitted.

3.1.4 Age ≥18 years.

3.1.5 Life expectancy ≥3 months.

3.1.6 ECOG performance status 0-2 / Karnofsky ≥60% (see **Appendix B**).

3.1.7 No minimum period since last therapy is defined, but patients must have adequate organ function not toxicity as defined in 3.1.8, and preexisting non-hematological dysfunction <grade 2.

3.1.8 Patients must have normal organ and marrow function as defined below:

- absolute neutrophil count ≥1.5 x 10^9^/l
- platelets ≥100 x 10^9^/l
- total bilirubin < 2 x upper limit of normal
- AST (SGOT) / ALT (SGPT) ≤2.5 x upper limit of normal
- creatinine ≤1.5 x upper limit of normal

3.1.9 Patients with known brain metastases may be included in this study if they fulfill all of the following criteria:

- the lesions have remained radiologically stable for at least six weeks after completion whole brain irradiation, and remain stable at the time of study entry
- there is no mass effect present radiologically
- the patient is no longer receiving steroids to control symptoms of brain metastases

3.1.10 Ability to understand and the willingness to sign a written informed consent document.

**3.2 Exclusion Criteria**

3.2.1 Patients with prior chemotherapy for metastatic melanoma.

3.2.2 Patients receiving any other investigational agents.

3.2.3 Uncontrolled intercurrent illness including, but not limited to, ongoing or active infection, uncontrolled hypertension, symptomatic congestive heart failure, unstable angina pectoris, cardiac arrhythmia, or psychiatric illness that would limit compliance with study requirements.

- - 1. Pregnant women are excluded from this study because the teratogenic or abortifacient effects of BAY 43-9006 are unknown.Women who are breastfeeding are not eligible or they must stop breastfeeding because of the unknown risk for adverse events in nursing infants.
    2. Patients with a history of serious allergic reactions to eggs (BAY 43-9006 is formulated using egg phospholipid).

3.2.6 Patients having any evidence of a bleeding diathesis. Patients on therapeutic anti-coagulation must have a weekly monitored PT/INR during the first cycle, then monitored closely thereafter as deemed necessary by the treating physician.

3.2.7 Patients who cannot swallow pills for whatever reason will be excluded.

3.2.8 HIV-positive patients receiving combination anti-retroviral therapy are excluded because of possible pharmacokinetic interactions with the investigational agent.

3.2.9 Patients with prior malignancies, except for adequately treated basal or squamous cell skin cancer, adequately treated non-invasive carcinomas or other cancers from which the patient has been disease-free for > 5 years.

**3.3 Inclusion of Women and Minorities**

*Both men and women and members of all ethnic groups are eligible for this trial, although accrual of non-white patients is expected to be low as melanoma is rare in non-white ethnic groups. Based on accrual in previous melanoma studies at NYU and Sydney, the proposed study population is illustrated in the table below:*

|  | **Race/Ethnicity** | | | | | |
| --- | --- | --- | --- | --- | --- | --- |
| **Gender** | White, not of Hispanic Origin | Black, not of Hispanic Origin | Hispanic | Asian or Pacific Islander | Unknown | Total |
| Male | 55% | 0 | 2.5 | 2.5 | 0 | 60% |
| Female | 35% | 0 | 2.5 | 2.5 | 0 | 40% |
| Total | 100% | 0 | 0 | 0 | 0 | 100% |

**4. TREATMENT PLAN**

**4.1** Patients will be stratified based on whether or not their tumors contain a B-RAF mutation. We will obtain either real time biopsies or paraffin embedded tissue from a previous biopsy for prospective identification of the B-RAF mutation.

**4.1.1 Agent Administration**

Treatment will be administered on an outpatient basis. Expected adverse events and appropriate dose modifications for BAY are described in Section 5. No investigational or commercial agents or therapies other than those described below may be administered with the intent to treat the patient’s malignancy.

All patients will keep a drug diary in order to record compliance with their drug regime.

(See Appendix H)

###### 4.1.2 BAY 43-9006

All patients will receive:

**BAY 43-9006 (NSC 724772) 400 mg po BID over 4 weeks.**

This dose may subsequently be reduced for individual patients, depending on toxicity (Section 5.2).

In order to study the effects of BAY 43-9006 on BRAF and on other components of the pathway, we will perform pretreatment and post-treatment tumor biopsies in the first 13 patients of each subgroup. The biopsies, which will be performed in the outpatient facility, will be either punch biopsies (6-8 mm diameter) or excisional biopsies performed by a surgical member of the Melanoma Disease Management team. The first portion of the biopsy will be used to evaluate the B-RAF status of the patient. Another portion of the tissue will be preserved in formalin and submitted to the NYU Department of Pathology. This material will be used to confirm the presence of melanoma and will be used for the immunohistochemical analyses of phospho-MAPK and cyclin D1 expression. The remaining portion will be trimmed of fat and excess connective tissue, and snap frozen in liquid N_2_. This material will be used for the Western blot analyses.

**4.2 Supportive Care Guidelines**

**4.2.1 Growth Factors**

Hematological growth factors will not be used prophylactically in this study, but may be used at the discretion of the investigator in the event of severe hematological toxicity.

**4.2.3 Contraception**

Women of childbearing potential and sexually active males are strongly advised to use an effective method of contraception.

- - 1. **Other**

Other appropriate supportive care medications may be administered at the investigator’s discretion. . In the event of hand-foot syndrome, patients may use topical emollients (such as Aquaphor), topical steroids, and/or anti-histamines for symptom relief.

**4.3 Duration of Therapy**

In the absence of treatment delays due to adverse event(s), treatment may continue until one of the following criteria applies:

- Disease progression,
- Intercurrent illness that prevents further administration of treatment,
- Unacceptable adverse event(s)
- Patient decides to withdraw from the study
- General or specific changes in the patient’s condition render the patient unacceptable for further treatment in the judgment of the investigator.
- If GI-Perforation (NOS), patients must be taken off therapy

1. **EXPECTED ADVERSE EVENTS / DOSE MODIFICATIONS**

5.1

- 1. **Expected Adverse Events Associated with BAY 43-9006**

**Comprehensive Adverse Events and Potential Risks List (CAEPR)**

**for**

**Sorafenib (BAY 43-9006, NSC 724772)**

The Comprehensive Adverse Event and Potential Risks list (CAEPR) provides a single, complete list of reported and/or potential adverse events (AE) associated with an agent using a uniform presentation of events by body system. In addition to the comprehensive list, a subset, the Agent Specific Adverse Event List (ASAEL), appears in a separate column and is identified with ***bold*** and ***italicized*** text. This subset of AEs (ASAEL) contains events that are considered 'expected' for expedited reporting purposes only. Refer to the 'CTEP, NCI Guidelines: Adverse Event Reporting Requirements' http://ctep.cancer.gov/reporting/adeers.html for further clarification. *Frequency is provided based on 1376 patients*. Below is the CAEPR for sorafenib.

**Comprehensive Adverse Events and Potential Risks List (CAEPR)**

**for**

**Sorafenib (BAY 43-9006, NSC 724772)**

The Comprehensive Adverse Event and Potential Risks list (CAEPR) provides a single list of reported and/or potential adverse events (AE) associated with an agent using a uniform presentation of events by body system. In addition to the comprehensive list, a subset, the Agent Specific Adverse Event List (ASAEL), appears in a separate column and is identified with ***bold*** and ***italicized*** text. This subset of AEs (ASAEL) contains events that are considered 'expected' for expedited reporting purposes only. Refer to the 'CTEP, NCI Guidelines: Adverse Event Reporting Requirements' http://ctep.cancer.gov/reporting/adeers.html for further clarification. *Frequency is provided based on 1376 patients*. Below is the CAEPR for sorafenib.

**Version 2.1, June 23, 2006^1^**

| **Adverse Events with Possible**  **Relationship to Sorafenib (BAY 43-9006)**  **(CTCAE v3.0 Term)**  **[n=1376 patients]** | | | **'Agent Specific Adverse Event List'**  **(ASAEL)** |
| --- | --- | --- | --- |
| **Likely (>20%)** | **Less Likely (<=20%)** | **Rare but Serious (<3%)** |
| ALLERGY/IMMUNOLOGY | | | |
|  | Allergic reaction/hypersensitivity (including drug fever) |  |  |
| BLOOD/BONE MARROW | | | |
|  | Hemoglobin |  |  |
|  | Leukocytes (total WBC) |  |  |
|  | Lymphopenia |  |  |
|  | Neutrophils/granulocytes (ANC/AGC) |  |  |
|  | Platelets |  |  |
| CARDIAC GENERAL | | | |
|  | Hypertension |  | ***Hypertension*** |
| CONSTITUTIONAL SYMPTOMS | | | |
| Fatigue (asthenia, lethargy, malaise) |  |  | ***Fatigue (asthenia, lethargy, malaise)*** |
|  | Fever (in the absence of neutropenia, where neutropenia is defined as ANC <1.0 x 10e9/L) |  |  |
|  | Rigors/chills |  |  |
|  | Weight loss |  |  |
| DERMATOLOGY/SKIN | | | |
|  | Dermatology/Skin - Other (non-life threatening squamous cell carcinoma of skin: keratoacanthoma type) |  |  |
|  | Dermatology/Skin - Other (other) |  |  |
|  | Dry skin |  |  |
|  | Flushing |  |  |
|  | Hair loss/alopecia (scalp or body) |  |  |
|  | Hypopigmentation |  |  |
|  | Nail changes |  |  |
|  | Pruritus/itching |  |  |
| Rash/desquamation |  |  | ***Rash/desquamation*** |
| Rash: hand-foot skin reaction |  |  | ***Rash: hand-foot skin reaction*** |
| GASTROINTESTINAL | | | |
|  | Anorexia |  |  |
|  | Ascites (non-malignant) |  |  |
|  | Constipation |  |  |
|  | Dehydration |  |  |
| Diarrhea |  |  | ***Diarrhea*** |
|  | Dysphagia (difficulty swallowing) |  |  |
|  | Flatulence |  |  |
|  | Heartburn/dyspepsia |  |  |
|  | Mucositis/stomatitis (functional/symptomatic): pharynx |  |  |
|  | Nausea |  |  |
|  |  | Perforation, GI - NOS |  |
|  | Vomiting |  |  |
| HEMORRHAGE/BLEEDING | | | |
|  | Hemorrhage GI - Select |  |  |
|  | Hemorrhage GU - Select |  |  |
| HEPATOBILIARY/PANCREAS | | | |
|  | Pancreatitis |  |  |
| INFECTION | | | |
|  | Febrile neutropenia (fever of unknown origin without clinically or microbiologically documented infection)(ANC <1.0 x 10e9/L, fever >=38.5 degrees C) |  |  |
|  | Infection with unknown ANC - Select |  |  |
| METABOLIC/LABORATORY | | | |
|  | Albumin, serum-low (hypoalbuminemia) |  |  |
|  | Alkaline phosphatase |  |  |
|  | ALT, SGPT (serum glutamic pyruvic transaminase) |  |  |
|  | Amylase |  |  |
|  | AST, SGOT (serum glutamic oxaloacetic transaminase) |  |  |
|  | Bilirubin (hyperbilirubinemia) |  |  |
|  | GGT (gamma-glutamyl transpeptidase) |  |  |
|  | Glucose, serum-high (hyperglycemia) |  |  |
|  | Lipase |  |  |
|  | Metabolic/Laboratory - Other (blood elastase) |  |  |
|  | Phosphate, serum-low (hypophosphatemia) |  |  |
| NEUROLOGY | | | |
|  | Neuropathy: sensory |  |  |
| PAIN | | | |
|  | Pain - abdomen NOS |  |  |
|  | Pain - joint |  |  |
|  | Pain - muscle |  |  |
|  | Pain NOS |  |  |
| PULMONARY/UPPER RESPIRATORY | | | |
|  | Hypoxia |  |  |
|  | Pleural effusion (non-malignant) |  |  |
|  | Pneumonitis/pulmonary infiltrates |  |  |
|  | Pneumothorax |  |  |
| RENAL/GENITOURINARY | | | |
|  | Renal failure |  |  |
| SYNDROMES | | | |
|  | Flu-like syndrome |  |  |

**^1^**This table will be updated as the toxicity profile of the agent is revised. Updates will be distributed to all Principal Investigators at the time of revision. The current version can be obtained by contacting ADEERSMD@tech-res.com. Your name, the name of the investigator, the protocol and the agent should be included in the e-mail.

**Also reported on BAY 43-9006 trials but with the relationship to BAY 43-9006 still undetermined:**

**ALLERGY/IMMUNOLOGY** - linear IgA disease

**CARDIAC ARRHYTHMIA** - atrial flutter; supraventricular arrhythmia

**CARDIAC GENERAL** - cardiac ischemia/infarction; left ventricular systolic dysfunction

**COAGULATION** - INR; PTT; thrombotic microangiopathy

**DERMATOLOGY/SKIN** - erythema multiforme

**GASTROINTESTINAL** - ileus

**HEMORRHAGE/BLEEDING** - CNS hemorrhage; petechiae; pleural hemorrhage; splenic infarction

**LYMPHATICS** - limb edema

**METABOLIC/LABORATORY** - creatinine; hyperuricemia; hyponatremia

**MUSCULOSKELETAL/SOFT TISSUE** - arthritis

**NEUROLOGY** - anxiety; CNS ischemia; dizziness; encephalopathy; memory impairment; psychosis; syncope

**OCULAR/VISUAL** - diplopia; uveitis

**PAIN** - back pain; bone pain; chest/thorax pain; headache; limb pain

**PULMONARY/UPPER RESPIRATORY** - ARDS; dyspnea; voice changes

**SEXUAL/REPRODUCTIVE FUNCTION** - erectile dysfunction

**VASCULAR** - thrombosis/thrombus/embolism

**Note**: BAY 43-9006 in combination with other agents could cause an exacerbation of any adverse event currently known to be caused by the other agent, or the combination may result in events never previously associated with either agent.

**Version 2.0, February 22, 2006^1^**

| **Adverse Events with Possible**  **Relationship to Sorafenib (BAY 43-9006)**  **(CTCAE v3.0 Term)**  **[n=1376 patients]** | | | | | | **'Agent Specific Adverse Event List'**  **(ASAEL)** |
| --- | --- | --- | --- | --- | --- | --- |
| **Likely (>20%)** | | **Less Likely (<=20%)** | **Rare but Serious (<3%)** | | |
| ALLERGY/IMMUNOLOGY | | | | | | |
|  | | Allergic reaction/hypersensitivity (including drug fever) |  | | |  |
| BLOOD/BONE MARROW | | | | | | |
|  | Hemoglobin | |  | |  | |
|  | Leukocytes (total WBC) | |  | |  | |
|  | Lymphopenia | |  | |  | |
|  | Neutrophils/granulocytes (ANC/AGC) | |  | |  | |
|  | Platelets | |  | |  | |
| CARDIAC GENERAL | | | | | | |
|  | | Hypertension | |  | | ***Hypertension*** |
| CONSTITUTIONAL SYMPTOMS | | | | | | |
| Fatigue (asthenia, lethargy, malaise) | |  | |  | | ***Fatigue (asthenia, lethargy, malaise)*** |
|  | | Fever (in the absence of neutropenia, where neutropenia is defined as ANC <1.0 x 10e9/L) | |  | |  |
|  | | Rigors/chills | |  | |  |
|  | | Weight loss | |  | |  |
| DERMATOLOGY/SKIN | | | | | | |
|  | | Dry skin | |  | |  |
|  | | Flushing | |  | |  |
|  | | Hair loss/alopecia (scalp or body) | |  | |  |
|  | | Hypopigmentation | |  | |  |
|  | | Nail changes | |  | |  |
|  | | Pruritus/itching | |  | |  |
| Rash/desquamation | |  | |  | | ***Rash/desquamation*** |
| Rash: hand-foot skin reaction | |  | |  | | ***Rash: hand-foot skin reaction*** |
|  | | Dermatology/skin – Other: non-life threatening squamous cell carcinoma of the skin: keratoacanthoma type | |  | |  |
| GASTROINTESTINAL | | | | | | |
|  | | Anorexia | |  | |  |
|  | | Ascites (non-malignant) | |  | |  |
|  | | Constipation | |  | |  |
|  | | Dehydration | |  | |  |
| Diarrhea | |  | |  | | ***Diarrhea*** |
|  | | Dysphagia (difficulty swallowing) | |  | |  |
|  | | Flatulence | |  | |  |
|  | | Heartburn/dyspepsia | |  | |  |
|  | | Mucositis/stomatitis (functional/symptomatic): pharynx | |  | |  |
|  | | Nausea | |  | |  |
|  | | Vomiting | |  | |  |
| HEMORRHAGE/BLEEDING | | | | | | |
|  | | Hemorrhage GI - Select | |  | |  |
|  | | Hemorrhage GU - Select | |  | |  |
| HEPATOBILIARY/PANCREAS | | | | | | |
|  | | Pancreatitis | |  | |  |
| INFECTION | | | | | | |
|  | | Febrile neutropenia (fever of unknown origin without clinically or microbiologically documented infection)(ANC <1.0 x 10e9/L, fever >=38.5 degrees C) | |  | |  |
|  | | Infection with unknown ANC-Select | |  | |  |
| METABOLIC/LABORATORY | | | | | | |
|  | | Albumin, serum-low (hypoalbuminemia) | |  | |  |
|  | | Alkaline phosphatase | |  | |  |
|  | | ALT, SGPT (serum glutamic pyruvic transaminase) | |  | |  |
|  | | Amylase | |  | |  |
|  | | AST, SGOT (serum glutamic oxaloacetic transaminase) | |  | |  |
|  | | Bilirubin (hyperbilirubinemia) | |  | |  |
|  | | GGT (gamma-glutamyl transpeptidase) | |  | |  |
|  | | Glucose, serum-high (hyperglycemia) | |  | |  |
|  | | Phosphate, serum-low (hypophosphatemia) | |  | |  |
|  | | Lipase | |  | |  |
|  | | Metabolic/Laboratory - Other (blood elastase) | |  | |  |
| NEUROLOGY | | | | | | |
|  | | Neuropathy: sensory | |  | |  |
| PAIN | | | | | | |
|  | | Pain - abdomen NOS | |  | |  |
|  | | Pain - joint | |  | |  |
|  | | Pain - muscle | |  | |  |
|  | | Pain NOS | |  | |  |
| PULMONARY/UPPER RESPIRATORY | | | | | | |
|  | | Hypoxia | |  | |  |
|  | | Pleural effusion (non-malignant) | |  | |  |
|  | | Pneumonitis/pulmonary infiltrates | |  | |  |
|  | | Pneumothorax | |  | |  |
| RENAL/GENITOURINARY | | | | | | |
|  | | Renal failure | |  | |  |
| SYNDROMES | | | | | | |
|  | | Flu-like syndrome | |  | |  |

**^1^**This table will be updated as the toxicity profile of the agent is revised. Updates will be distributed to all Principal Investigators at the time of revision. The current version can be obtained by contacting ADEERSMD@tech-res.com. Your name, the name of the investigator, the protocol and the agent should be included in the e-mail.

**Also reported on sorafenib trials but with the relationship to sorafenib still undetermined:**

**ALLERGY/IMMUNOLOGY** - linear IgA disease

**CARDIAC ARRHYTHMIA** - atrial flutter; supraventricular arrhythmia

**CARDIAC GENERAL** - cardiac ischemia/infarction; left ventricular systolic dysfunction

**COAGULATION** - INR; PTT; thrombotic microangiopathy

**DERMATOLOGY/SKIN** – erythema multiforme

**GASTROINTESTINAL** - ileus

**HEMORRHAGE/BLEEDING** - CNS hemorrhage; petechiae; pleural hemorrhage; splenic infarction

**LYMPHATICS** - limb edema

**METABOLIC/LABORATORY** - creatinine; hyperuricemia; hyponatremia

**MUSCULOSKELETAL/SOFT TISSUE** - arthritis

**NEUROLOGY** - anxiety; CNS ischemia; dizziness; encephalopathy; memory impairment; psychosis; syncope

**OCULAR/VISUAL** - diplopia; uveitis

**PAIN** - back pain; bone pain; chest/thorax pain; headache; limb pain

**PULMONARY/UPPER RESPIRATORY** - ARDS; dyspnea; voice changes

**SEXUAL/REPRODUCTIVE FUNCTION –** erectile dysfunction

**VASCULAR** - thrombosis/thrombus/embolism

**Note**: Sorafenib in combination with other agents could cause an exacerbation of any adverse event currently known to be caused by the other agent, or the combination may result in events never previously associated with either agent.

**5.2 Dosing Delays / Dose Modifications**

Prior to re-treatment, patients must have recovered the following organ function:

- absolute neutrophil count ≥1.0 x 10^9^/l
- platelets ≥100 x 10^9^/l
- total bilirubin < 2 x upper limit of normal
- AST (SGOT) / ALT (SGPT) ≤2.5 x upper limit of normal
- creatinine ≤1.5 x upper limit of normal
- other Grade 0-1
- Patients should be dose reduced to 400mg po in AM and 200mg po in PM in the event of any of the following:
- Nausea or vomiting dose reductions should only be in the face of maximal anti-emetic therapy
- Grade 4 non-hematologic toxicity: if study drug related patients should be taken off study
- Grade 3 non-hematologic toxicity: hold BAY43-9006 and reevaluate patient weekly until toxicity improves to < Grade 1. Reduce dose by 200 mg /day and no more than 2 dose reductions allowed.
- Increased liver functions (Grade 3-4): withhold treatment until resolved to < grade 1 toxicity and decrease by 200 mg.
- Grade 4 hematologic toxicity: withhold treatment until resolved to < grade 1 toxicity and decrease by 200 mg. If Grade 4 neutropenia lasts for more than 7 days after therapy with growth factor support contact PI.
- Many patients will have a rash that will require drug withdrawal and toxicity resolution before restarting. Hand-foot syndrome will require drug withdrawal and reduction

Management of Treatment-emergent Hypertension:

| **Grade of Event (CTCAE v.3)** | **Management/ Next Dose** |
| --- | --- |
| grade 1 | Consider increased BP monitoring |
| grade 2 asymptomatic and diastolic BP < 110 mm Hg | Begin anti-hypertensive therapy and continue agent |
| grade 2 symptomatic/ persistent  OR  diastolic BP ≥ 110 mm Hg  OR  grade 3 | 1. Agent should be held* until symptoms resolve  and diastolic BP ≤ 100 mm Hg; also  treat patient with anti-hypertensives and when  agent is restarted, reduce by 1 dose level.**  2. If diastolic BP not controlled (≤ 100) on  therapy, reduce another dose level *** |
| grade 4 | Discontinue protocol therapy |
| * Patients requiring a delay of > 2 weeks should go off protocol therapy.  ** May be able to resume full dose later.  *** Patients requiring > 2 dose reductions should go off protocol therapy. | |

Current CTCAE definitions used by CTEP:

Grade 1: asymptomatic, transient (< 24 hours) increase by > 20 mmHg (diastolic) or to

>150/100 if previously WNL; intervention not indicated

Grade 2: recurrent or persistent (> 24 hours) or symptomatic increase by > 20 mmHg

(diastolic) or to > 150/100 if previously WNL; monotherapy may be indicated

Grade 3: requiring more than one drug or more intensive therapy than previously

Grade 4: life threatening (e.g. hypertensive crisis)

Patients will be allowed 3 weeks for resolution of toxicity. Treatment will be discontinued if any grade 3 or 4 toxicity does not resolve in 3 weeks.

No patients should have his / her dose re-escalated following dose reduction.

**6. AGENT FORMULATION AND PROCUREMENT**

**6.1 Study Agent**

# BAY 43-9006 tosylate (NSC 724772)

**Chemical Name:** 4–{4-[3-(4-chloro-3-trifluoromethyl-phenyl) ureido]-phenoxy}-pyridine-2 carboxylic acid methylamide-4-methylbenzensulfonate.

**Other Names:** BAY 54-9085 is the tosylate salt of BAY 43-9006; sorafenib

**Classification:** Kinase inhibitor (Raf, VEGF-R, and PDGF-R)

**Mechanism of Action:**The ras/raf signaling pathway is an important mediator of responses to growth signals and angiogenic factors. This pathway is often aberrantly activated in human tumors due to presence of activated ras, mutant b-raf, or over expression of growth factor receptors.

BAY 43-9006 is a potent inhibitor of c-raf, and wild-type and mutant b-raf in vitro. Additionally, further characterization of BAY 43-9006 tosylate revealed that this agent inhibits several receptor tyrosine kinases (RTKs) that are involved in tumor progression (VEGF-R, PDGF-R, Flt3, and c-KIT) and p38α, a member of the MAPK family.

**Molecular Formula:** C_12_H_16_CIF_3_N_4_O_3_ X C_7_H_8_O_3_S

**M.W.:** BAY 43-9006 tosylate: 637 Daltons; BAY 43-9006 (free base): 465 Daltons

**Approximate Solubility:** 0.19 mg/100 mL in 0.1 N HCl, 453 mg/100 mL in Ethanol, and 2971 mg/100 mL in PEG 400.

**How Supplied:** BAY 43-9006 tosylate is supplied as an immediate-release film-coated, round, and salmon color tablet containing 200 mg of the free base, BAY 43-9006, and the excipients croscarmellose sodium, microcrystalline cellulose, hydroxypropylmethylcellulose, sodium lauryl sulfate, and magnesium stearate. The film-coat consists of hydroxypropylmethyl cellulose, polyethylene glycol, tiranium dioxide and red iron oxide. The film coating has no effect on the rate of release of the active BAY 43-9006 tosylate.

BAY 43-9006 tosylate 200 mg tablets are supplied in bottles of 140 tablets.

**Storage:** Store at controlled room temperature (15ºC – 25ºC). Storage conditions should not exceed 25ºC.

**Stability:** Stability studies with the 200 mg dosage form are ongoing. The current shelf life is 24 months when stored at controlled room temperature.

**Route**(s) **of Administration:** Orally

**Method of Administration:** BAY 43-9006 tosylate should be taken with at least 250 mL of water and can be given without regards to meals. Food does not appear to have a clear effect on BAY 43 9006 tosylate pharmacokinetics.

**Potential Drug Interactions:** BAY-9006 tosylate is metabolized by the P450 CYP3A enzyme and has been shown in preclinical studies to inhibit multiple CYP isoforms. Therefore, it is possible that BAY-9006 tosylate may interact with drugs that are metabolized by the P450 CYP isoenzymes or with drugs that inhibit CYP 3A. Close monitoring is recommended for patients taking agents with narrow therapeutic indices and metabolized by the liver, such as warfarin, phenytoin, quinidine, carbamazepine, phenobarbital, cyclosporine, and digoxin. Additionally, BAY-9006 tosylate is 97% to 99% protein bound; however, no drug interactions have been reported in studies, thus far.

**6.2 Agent Ordering**

Except in very unusual circumstances, each Participating Institution will order DCTD-supplied investigational agents directly from CTEP. Investigational agents may be ordered by a participating site only after the initial IRB approval for the site has been forwarded by the Coordinating Center to the CTEP PIO.

NCI-supplied agents may be requested by the Protocol Chair (or their authorized designees) at each Participating Institution. Pharmaceutical Management Branch (PMB) policy requires that the agent be shipped directly to the institution where the patient is to be treated. PMB does not permit the transfer of agents between institutions (unless prior approval from PMB is obtained). Completed Clinical Drug Requests (NIH-986) should be submitted to the PMB by fax (301) 480-4612 or mailed to the Pharmaceutical Management Branch, CTEP, DCTD, NCI, 9000 Rockville Pike, EPN Rm. 7149, Bethesda, MD 20892.

**6.3 Agent Accountability**

The Investigator, or a responsible party designated by the investigator, must maintain a careful record of the inventory and disposition of all agents received from DCTD using the NCI Drug Accountability Record Form.

**7.0 CORRELATIVE/SPECIAL STUDIES**

**7.1** *BRAF* exon 15 and *NRAS* exon 3 will be sequenced from paraffin-embedded melanoma tumor tissues by the Osman/ Polsky lab at NYU. In our published study, 92% of mutations in metastatic melanoma tissues were in BRAF exon 15. BRAF exon 11 will not be examined before treatment because our study indicated that only (2/77) 2.6% of metastatic melanoma tissues harbor this mutation. (Cancer Res. 2003 Jul 15;63(14):3955-7.) NRAS will be assessed despite its relatively rare occurrence (exon 3 mutations were found in only 4% of tumors in our study) because it functions upstream of BRAF. Activating mutations in NRAS, therefore, may lead to an increase in the level of BRAF activity and may explain why a patient with a wild-type BRAF tumor would response to treatment with BAY 43-9006.

We expect tumors from 6 or 7 patients to harbor a *BRAF* mutation and tumors from 1 or 2 patients to harbor an *NRAS* mutation. We anticipate that 2-3 patients will have no detectable mutation in either of these genes.

Representative tumor tissue sections will be sent to the Osman/Polsky laboratory at NYU via hand delivery or overnight mail to ensure timely delivery. Genomic DNA will be extracted from paraffin tissue blocks using a Qiagen Tissue Kit (Qiagen, Valencia, CA). Exon 3 of N-RAS and exon 15 of BRAF will be amplified by PCR and sequenced using the methods described in our published paper. Turn-around time is expected to be within 10 days of receipt of tumor samples. (**Appendix C**)

**7.2** Tumor levels of CDK4, phospho-MAPK, and cyclin D1 will be assessed by our newly optimized quantitative immunohistochemistry. Expression of p85 PI3 kinase (expected to be unaffected by treatment) will be assessed as internal controls. (**Appendix D**).

These studies will be done using the formalin-fixed portion of the biopsies. By utilizing quantitative immunohistochemistry to determine relative alterations in protein expression levels, we eliminate the need for handling and delivery of frozen tissue. In addition, we will measure phosphorylated MAPK by immunohistochemistry using an antibody to the activated, phosphorylated MAPK (Cell Signaling Technology, Beverly, MA). These studies will be done using the formalin- fixed portion of the biopsies. As a result, these analyses will not exhaust tissues required for the immunoblotting studies described above.(**Appendix D**)

**7.3** We will use a fluorescent, mutant-specific PCR-based approach to detect circulating tumor cells that harbor the most common *BRAF* mutation (T1796A). For each patient, we will correlate our results with the presence of *BRAF* mutation in their tumor tissues, as well as their response to treatment (**See Appendix E**).

- 1. A variety of studies have suggested that cellular interactions with the Extracellular Matrix (ECM) can regulate a wide variety of cellular functions including adhesion, migration and proliferation (31). Angiogenesis, malignant tumor growth and metastasis all require proteolytic remodeling of components of extracellular matrix (32). Interestingly, three studies have suggested that cellular interactions with the Extracellular Matrix (ECM) can regulate a wide variety of cellular functions including adhesion, migration and proliferation (31). Angiogenesis, malignant tumor growth and metastasis all require proteolytic remodeling of components of extracellular matrix (32). Interestingly, recent studies have identified cryptic regulatory epitopes that are normally hidden within the three dimensional structure of the ECM protein collagen (33), (34). Under normal physiological conditions these cryptic epitopes of collagen are not exposed (33), (34). However, following proteolytic remodeling of the collagenous ECM during tumor growth and invasion these unique cryptic epitopes are exposed and can be recognized by two novel monoclonal antibodies termed HUIV26 and HUI77 (33), (34). The functional cryptic epitopes defined by these unique Mabs have been shown to be specifically associated with angiogenic blood vessels and proliferating tumors in vivo, while little if any was detected within normal tissues (33), (34). These findings suggest that exposure of these epitopes may correlate with induction of angiogenesis and tumor progression (33), (34). Interestingly, during collagen synthesis pro-peptide segments of the collagen chains are cleaved and released into the circulation as part of the collagen fibril formation. In fact, investigators have developed an ELISA based assay to quantify the levels of collagen pro-peptide and found a correlation with tumor growth and metastasis (35), (36). Based on these studies, the possibility that Peter Brooks laboratory’s unique cryptic epitopes of collagen could also be cleaved and released into the systemic circulation was tested. Brooks and colleagues have developed an ELISA based assay to detect soluble HUIV26 and HUI77 cryptic epitopes that is based upon a sandwich assay employing the use of a biotinylated anti-type IV collagen polyclonal antibody along with an anti-biotin monoclonal antibody conjugated to horseradish peroxidase as depicted in Figure 7. This assay has been subjected to an extensive validation regimen and as shown in Figure 8 and has the necessary range of linearity and sensitivity to allow it to be amenable for the monitoring of a clinical protocol where serial samples will be monitored. Figure 9 also demonstrates the results of preliminary findings which indicate that the cryptic epitopes of collagen can be detected in serum from melanoma patients. Importantly, the levels of these cryptic epitopes were significantly elevated in serum from melanoma patients as compared to serum from normal subjects. These findings suggest the possibility that the levels of cryptic collagen epitopes may correlate with extent of tumor burden, invasion and metastasis. Thus, serum levels of these unique cryptic epitopes may be useful to monitor the course of diseases following therapeutic intervention. We will examine levels over the duration of treatment and will examine the relationship with the extent of tumor burden. (**Appendix F**)


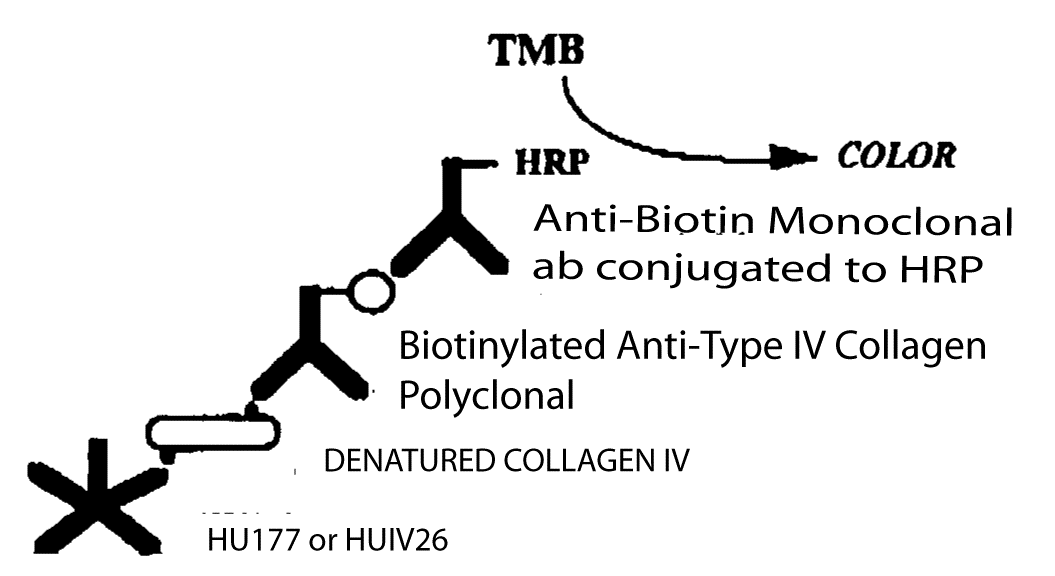


# Figure 7. Key elements of ELISA assay to detect HUIV26 and HUI77 cryptic epitopes of collagen.


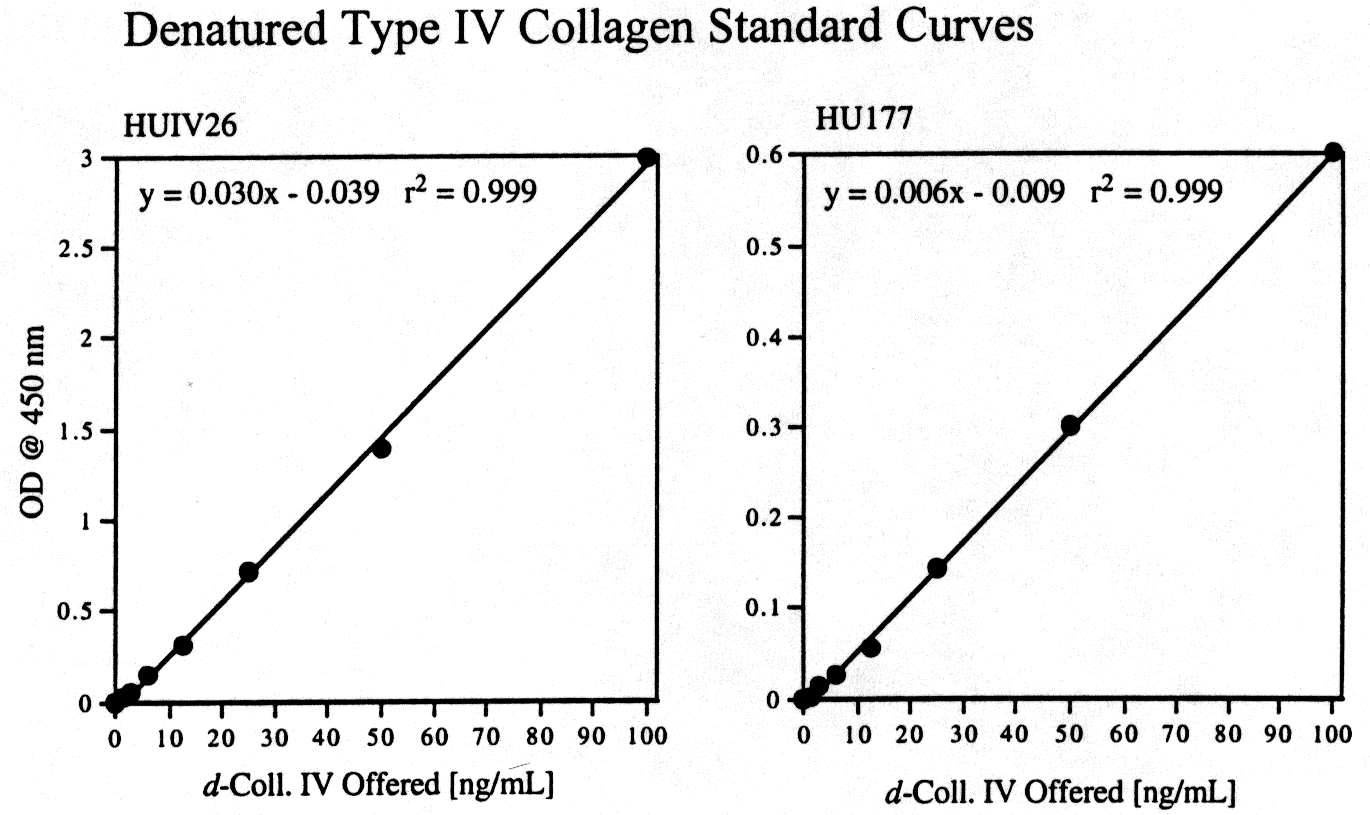


# Figure 8. Representative standard curves showing the linear range of the ELISA assay for the detection of HUIV26 and HUI77 collagen cryptic epitopes in human serum.


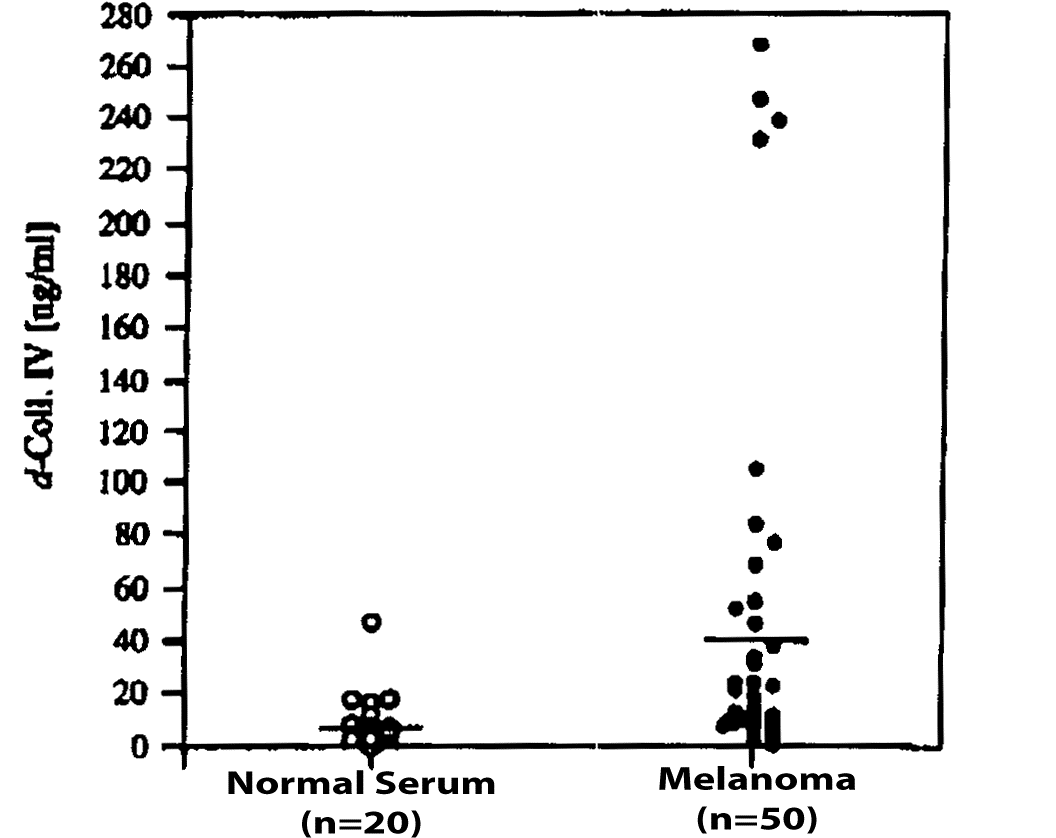


**Figure 9**. Scatter graph showing the cluster of denatured TYPE IV collagen cryptic epitope values in serum from melanoma patients compared to normal controls using HU177 antibody.

**8. STUDY CALENDAR**

Baseline evaluations are to be conducted no more than 1 week prior to administration of study agent. Scans and x-rays must be done no more than 4 weeks prior to the start of therapy. In the event that the patient’s condition is deteriorating, laboratory evaluations should be repeated no more than 48 hours prior to initiation of the next cycle of therapy.

|  | **Pre-Study (<14 days before starting drug)** | **Each cycle** | | | **Every 2 cycles** | **Off Study** |
| --- | --- | --- | --- | --- | --- | --- |
|  |  | **D1** | **D8*,15*,22*** | **D28** |  |  |
| BAY 43-9006 |  | X | X | X |  |  |
| **Informed consent** | X |  |  |  |  |  |
| **Demographics** | X |  |  |  |  |  |
| **Medical history** | X |  |  |  |  |  |
| **Physical exam** | X | X |  | X |  | X |
| **Vital signs** | X | X | X | X |  | X |
| **Height / Weight** | X | X |  |  |  | X |
| **Medications** | X | X |  |  |  | X |
| **Performance status** | X | X |  | X |  | X |
| **Hb, ANC, plts** | X | X |  |  |  | X |
| **Serum chemistry**** | X | X |  |  |  | X |
| **EKG** | X |  |  |  |  |  |
| **B-HCG** | X |  |  |  |  |  |
| **Adverse Events** |  | X |  |  |  | X |
| **Tumor Measurements** |  | X |  | X^1^ | X^2^ | X |
| **Collection of PBPC’s** |  | X |  | X |  | X |
| **Collagen Cryptic Epitopes** |  | X |  | X |  |  |
| **Tumor Biopsy** |  | X^***^ |  | X^***†^ |  |  |
| **PT/INR if on therapeutic anti-coagulation** | X | X | X | X |  | X |

* Weekly blood pressures will be done for the first cycle.

**Electrolytes, calcium, magnesium, liver function tests (AST, ALT, total bili) and LDH.

*** Biopsies done only on Cycle 1.

† The post treatment tumor biopsy will be conducted 18-48 hours after the first treatment cycle.

^1^ Day 28 tumor measurements will be based on the measurement of tumors sites that can be assessed by clinical exam.

^2^ The every other cycle, day 28 tumor measurements will be assessed by imaging studies.

**9.0 MEASUREMENT OF EFFECT**

For the purposes of this study, patients should be reevaluated for response every 2 cycles. In addition to a baseline scan, confirmatory scans should also be obtained *not less than 4* weeks following initial documentation of objective response.

**9.1 Definitions**

Response and progression will be evaluated in this study using the new international criteria proposed by the Response Evaluation Criteria in Solid Tumors (RECIST) Committee [*JNCI* 92(3):205-216, 2000]. Changes in only the largest diameter (unidimensional measurement) of the tumor lesions are used in the RECIST criteria. Note: Lesions are either measurable or non-measurable using the criteria provided below.

**9.1.1 Measurable disease**

Measurable lesions are defined as those that can be accurately measured in at least one dimension (longest diameter to be recorded) as ≥20 mm with conventional techniques (PET, CT, MRI, x-ray) or as ≥10 mm with spiral CT scan. All tumor measurements must be recorded in millimeters.

**Note:** Tumor lesions that are situated in a previously irradiated area will be considered measurable only if there has been documented progression of the lesion since completion of radiotherapy.

Clinical lesions

Clinical lesions will only be considered measurable when they are superficial (e.g., skin nodules and palpable lymph nodes). In the case of skin lesions, documentation by color photography, including a ruler to estimate the size of the lesion, is recommended.

Chest x-ray

Lesions on chest x-ray are acceptable as measurable lesions when they are clearly defined and surrounded by aerated lung. However, CT is preferable.

Conventional CT and MRI

These techniques should be performed with cuts of 10 mm or less in slice thickness contiguously. Spiral CT should be performed using a 5 mm contiguous reconstruction algorithm. This applies to tumors of the chest, abdomen, and pelvis. Head and neck tumors and those of extremities usually require specific protocols.

Ultrasound (US)

When the primary endpoint of the study is objective response evaluation, US should not be used to measure tumor lesions. It is, however, a possible alternative to clinical measurements of superficial palpable lymph nodes, subcutaneous lesions, and thyroid nodules. US might also be useful to confirm the complete disappearance of superficial lesions usually assessed by clinical examination.

Endoscopy, Laparoscopy

The utilization of these techniques for objective tumor evaluation has not yet been fully and widely validated. Their uses in this specific context require sophisticated equipment and a high level of expertise that may only be available in some centers. Therefore, the utilization of such techniques for objective tumor response should be restricted to validation purposes in reference centers. However, such techniques may be useful to confirm complete pathological response when biopsies are obtained.

Cytology, Histology

These techniques can be used to differentiate between partial responses (PR) and complete responses (CR) in rare cases (e.g., residual lesions in tumor types, such as germ cell tumors, where known residual benign tumors can remain).

The cytological confirmation of the neoplastic origin of any effusion that appears or worsens during treatment when the measurable tumor has met criteria for response or stable disease is mandatory to differentiate between response or stable disease (an effusion may be a side effect of the treatment) and progressive disease.

**9.1.2 Non-measurable disease**

All other lesions (or sites of disease), including small lesions (longest diameter <20 mm with conventional techniques or <10 mm using spiral CT scan), are considered non-measurable disease. Bone lesions, leptomeningeal disease, ascites, pleural/pericardial effusions, lymphangitis cutis/pulmonis, inflammatory breast disease, abdominal masses (not followed by CT or MRI), and cystic lesions are all non-measurable.

**9.1.3 Target lesions**

All measurable lesions up to a maximum of five lesions per organ and 10 lesions in total representative of all involved organs should be identified as **target lesions** and recorded and measured at baseline. Target lesions should be selected on the basis of their size (lesions with the longest diameter) and their suitability for accurate repeated measurements (either by imaging techniques or clinically). A sum of the longest diameter (LD) for all target lesions will be calculated and reported as the baseline sum LD. The baseline sum LD will be used as reference by which to characterize the objective tumor response.

**9.1.4 Non-target lesions**

All other lesions (or sites of disease) should be identified as **non-target lesions** and should also be recorded at baseline. Non-target lesions include measurable lesions that exceed the maximum numbers per organ or total of all involved organs as well as non-measurable lesions. Measurements of these lesions are not required, but the presence or absence of each should be noted throughout follow-up.

**9.2 Guidelines for Evaluation of Measurable Disease**

All measurements should be taken and recorded in metric notation using a ruler or calipers. All baseline evaluations should be performed as closely as possible to the beginning of treatment and never more than 4 weeks before the beginning of the treatment.

The same method of assessment and the same technique should be used to characterize each identified and reported lesion at baseline and during follow-up. Imaging-based evaluation is preferred to evaluation by clinical examination when both methods have been used to assess the antitumor effect of a treatment.

**9.3** **Response Criteria**

**9.3.1 Evaluation of target lesions**

Complete Response (CR)

Disappearance of all target lesions

Partial Response (PR)

At least a 30% decrease in the sum of the longest diameter (LD) of target lesions, taking as reference the baseline sum LD

Progressive Disease (PD)

At least a 20% increase in the sum of the LD of target lesions, taking as reference the smallest sum LD recorded since the treatment started or the appearance of one or more new lesions

Stable Disease (SD)

Neither sufficient shrinkage to qualify for PR nor sufficient increase to qualify for PD, taking as reference the smallest sum LD since the treatment started

**9.3.2 Evaluation of non-target lesions**

Complete Response (CR)

Disappearance of all non-target lesions and normalization of tumor marker level

Note: If tumor markers are initially above the upper normal limit, they must normalize for a patient to be considered in complete clinical response.

Incomplete Response/Stable Disease (SD)

Persistence of one or more non-target lesion(s) and/or maintenance of tumor marker level above the normal limits

Progressive Disease (PD)

Appearance of one or more new lesions and/or unequivocal progression of existing non-target lesions

Although a clear progression of “non-target” lesions only is exceptional, in such circumstances the opinion of the treating physician should prevail, and the progression status should be confirmed at a later time by the review panel (or study chair).

**9.3.3 Evaluation of best overall response**

The best overall response is the best response recorded from the start of the treatment until disease progression/recurrence (taking as reference for progressive disease the smallest measurements recorded since the treatment started). The patient's best response assignment will depend on the achievement of both measurement and confirmation criteria (see section 9.3.1).

| Target Lesions | Non-Target Lesions | New Lesions | Overall Response |
| --- | --- | --- | --- |
| CR | CR | No | CR |
| CR | Incomplete response/SD | No | PR |
| PR | Non-PD | No | PR |
| SD | Non-PD | No | SD |
| PD | Any | Yes or No | PD |
| Any | PD | Yes or No | PD |
| Any | Any | Yes | PD |

**Note:**

- Patients with a global deterioration of health status requiring discontinuation of treatment without objective evidence of disease progression at that time should be classified as having “symptomatic deterioration.” Every effort should be made to document the objective progression, even after discontinuation of treatment.
- In some circumstances, it may be difficult to distinguish residual disease from normal tissue. When the evaluation of complete response depends on this determination, it is recommended that the residual lesion be investigated (fine needle aspirate/biopsy) before confirming the complete response status.

**9.4 Confirmatory Measurement / Duration of Response**

**9.4.1 Confirmation**

To be assigned a status of PR or CR, changes in tumor measurements must be confirmed by repeat assessments that should be performed no less than 4 weeks after the criteria for response are first met. In the case of SD, follow-up measurements must have met the SD criteria at least once after study entry at a minimum interval of 6 weeks (see section 9.3.3). Assessments are made every 56 days.

**9.4.2 Duration of overall response**

The duration of overall response is measured from the time measurement criteria are met for CR or PR (whichever is first recorded) until the first date that recurrent or progressive disease is objectively documented (taking as reference for progressive disease the smallest measurements recorded since the treatment started).

The duration of overall CR is measured from the time measurement criteria are first met for CR until the first date that recurrent disease is objectively documented.

**9.4.3 Duration of stable disease**

Stable disease is measured from the start of the treatment until the criteria for progression are met, taking as reference the smallest measurements recorded since the treatment started.

**9.5 Time to Progression**

Time to progression is defined as the time from the first day of treatment with BAY until the first documentation of disease progression.

**9.6**  **Response Review**

All responses will be reviewed by an expert(s) independent of the study at the study’s completion. Simultaneous review of the patients’ files and radiological images will be performed.

**10. REGULATORY AND REPORTING REQUIREMENTS**

- 1. **Patient Registration**

Prior to registration, each Participating Institution must forward a copy of its IRB approval and approved consent form to the Coordinating Center (Montefiore Medical Center). The Coordinating Center will send IRB approvals to the NCI Protocol Information Office (see Multicenter Guidelines, section 10.4).

To register a patient, a completed enrollment form, eligibility checklist and signed consent form should be sent to the Coordinating Office (Montefiore Hospital). These forms may be faxed to Lisa Escobar at the Coordinating Center 718 822-0335

Following central eligibility verification, registration will be confirmed by allocation of a patient identification number.

**10.2 Adverse Event Reporting**

- - 1. **Common Toxicity Criteria**

Adverse events (AE) will use the descriptions and grading scales found in the revised NCI Common Terminology Criteria for Adverse Events (CTCAE). This study will utilize the CTCEA version 3.0 for adverse event reporting. All appropriate treatment areas should have access to a copy of the CTCAE version 3.0, which is available at <http://ctep.info.nih.gov/CTC3/>.

- - 1. **Expedited Reporting Guidelines**

**EXPEDITED ADVERSE EVENT REPORTING**

**PHASE II AND III STUDIES WITH INVESTIGATIONAL AGENTS**

**SUPPLIED UNDER CTEP-SPONSORED IND’S**

**Phase 2 and 3 Trials Utilizing an Agent under a CTEP IND: AdEERS Reporting Requirements for Adverse Events That Occur Within 30 Days^1^ of the Last Dose of the Investigational Agent**

|  | **Grade 1** | **Grade 2** | **Grade 2** | **Grade 3** | | **Grade 3** | | **Grades**  **4 & 5^2^** | **Grades**  **4 & 5^2^** |
| --- | --- | --- | --- | --- | --- | --- | --- | --- | --- |
|  | **Unexpected**  **and Expected** | **Unex-pected** | **Expected** | **Unexpected** | | **Expected** | | **Unex-**  **pected** | **Expected** |
|  |  |  |  | **with**  **Hospitali-zation** | **without Hospitali- zation** | **with**  **Hospitali-**  **zation** | **without Hospitali-**  **zation** |  |  |
| **Unrelated**  **Unlikely** | Not  Required | Not  Required | Not  Required | 10 Calendar  Days | Not  Required | 10 Calendar  Days | Not  Required | 10  Calendar  Days | 10  Calendar  Days |
| **Possible**  **Probable**  **Definite** | Not  Required | 10 Calendar  Days | Not  Required | 10 Calendar  Days | 10 Calendar  Days | 10 Calendar  Days | Not  Required | 24-Hour;  5 Calendar  Days | 10  Calendar  Days |
| **^1^ Adverse events with attribution of possible, probable, or definite that occur greater than 30 days after the last dose of treatment with an agent under a CTEP IND require reporting as follows:**  AdEERS 24-hour notification followed by complete report within 5 calendar days for:   - Grade 4 and Grade 5 unexpected events   AdEERS 10 calendar day report:   - Grade 3 unexpected events with hospitalization or prolongation of hospitalization - Grade 5 expected events   ^2^ Although an AdEERS 24-hour notification is not required for death clearly related to progressive disease, a full report is required as outlined in the table.  December 15, 2004 | | | | | | | | | |

**Note:  All deaths on study require both routine and expedited reporting regardless of causality.  Attribution to treatment or other cause must be provided.**

- Expedited AE reporting timelines defined:
  - - “24 hours; 5 calendar days” – The investigator must initially report the AE via AdEERS within 24 hours of learning of the event followed by a complete AdEERS report within 5 calendar days of the initial 24-hour report.
    - “10 calendar days” - A complete AdEERS report on the AE must be submitted within 10 calendar days of the investigator learning of the event.
- Any medical event equivalent to CTCAE grade 3, 4, or 5 that precipitates hospitalization (or prolongation of existing hospitalization) must be reported regardless of attribution and designation as expected or unexpected with the exception of any events identified as protocol-specific expedited adverse event reporting exclusions.
- Any event that results in persistent or significant disabilities/incapacities, congenital anomalies, or birth defects must be reported via AdEERS if the event occurs following treatment with an agent under a CTEP IND.
- Use the NCI protocol number and the protocol-specific patient ID provided during trial registration on all reports.

A table showing the expected adverse events associated with BMS 247550 can be found in section 5.1 and in Appendix A.

- Telephone reports to the Investigational Drug Branch at 1 (301) 230 2330 available 24 hours daily (recorder between 5pm and 9am EST).
- Expedited reports are to be submitted using AdEERS or the paper templates available at [http://ctep.info.nih.gov](http://ctep.info.nih.gov/). The NCI guidelines for expedited adverse event reporting are also available at this site.
- A list of agent specific expected adverse events (ASAEL) can be found in this protocol.

**10.2.3 Forms**

AE’s should be reported electronically using the AdEERS system, full details of which are available at <http://ctep.info.nih.gov/AdEERS/>.

If electronic reporting is not possible within the required timeframe, reports should be made on the DCTD Form for Reporting AEs Occurring with Investigational Agents Obtained from the NCI. This form can be downloaded from the CTEP home page <http://ctep.info.nih.gov/InfoForms/default.htm>. Cases of secondary AML/MDS are to be reported using the NCI/CTEP Secondary AML/MDS Report Form.

**10.3 Data Reporting**

Data will be reported electronically to the Coordinating Center, using an Excel spreadsheet template that will be provided to the Participating Institutions.

Data updates should be forwarded to the Coordinating Center within 2 weeks of completion of each treatment cycle.

This study will be monitored by the Clinical Data Update System (CDUS) version 2.0. Cumulative CDUS data will be submitted electronically to CTEP by the Coordinating Center on a quarterly basis. Reports are due January 31, April 30, July 31, and October 31. Instructions for submitting data using the CDUS can be found on the CTEP home page (<http://ctep.info.nih.gov/CtepInformatics/CDUS/Default.htm>). Sample data collection forms are contained in Appendix G.

**10.4 CTEP Multicenter Guidelines**

**10.4.1 Responsibilities of the Protocol Chair**

- The Protocol Chair will be the single liaison with the CTEP Protocol and Information Office (PIO). The Protocol Chair is responsible for the coordination, development, submission, and approval of the protocol as well as its subsequent amendments. The protocol must not be rewritten or modified by anyone other than the Protocol Chair. There will be only one version of the protocol, and each Participating Institution will use that document. The Protocol Chair is responsible for assuring that all Participating Institutions are using the correct version of the protocol.
- The Protocol Chair is responsible for the overall conduct of the study at all Participating Institutions and for monitoring its progress. All reporting requirements to CTEP are the responsibility of the Protocol Chair.
- The Protocol Chair is responsible for the timely review of Adverse Events (AE) to assure safety of the patients.
- The Protocol Chair will be responsible for the review of and timely submission of data for study analysis.

**10.4.2 Responsibilities of the Coordinating Center**

- Each Participating Institution will have an appropriate assurance on file with the Office for Protection from Research Risks (OPRR), NIH. The Coordinating Center is responsible for assuring that each Participating Institution has an OPRR assurance and must maintain copies of IRB approvals from each participating site.
- Prior to the activation of the protocol at each Participating Institution, an OPRR form 310 (documentation of IRB approval) must be submitted to the CTEP PIO.
- The Coordinating Center is responsible for central patient registration. The Coordinating Center is responsible for assuring that IRB approval has been obtained at each participating site prior to the first patient registration from that site.
- The Coordinating Center is responsible for the preparation of all submitted data for review by the Protocol Chair.
- The Coordinating Center will maintain documentation of AE reports. There are two options for AE reporting: (1) Participating Institutions may report directly to CTEP with a copy to the Coordinating Center, or (2) Participating Institutions report to the Coordinating Center who in turn report to CTEP. The Coordinating Center will submit AE reports to the Protocol Chair for timely review.
- Audits may be accomplished in two ways: (1) source documents and research records for selected patients are brought from participating sites to the Coordinating Center for NCI audit , and (2) selected patient records will be audited on-site at participating sites yearly. The NCI will be perform all audits at the Coordinating Center, subsequently the Coordinating Center will be responsible for having all source documents, research records, all IRB approval documents, NCI Drug Accountability Record forms, patient registration lists, response assessments scans, x-rays, etc. available for the audit.

**10.5 Clinical Trials Agreement (CTA)**

The agent, BAY 43-9006, used in this protocol is provided to the NCI under a Clinical Trials Agreement (CTA) between Bayer Pharmaceuticals [hereinafter referred to as “Collaborator”] and the NCI Division of Cancer Treatment and Diagnosis. Therefore, the following obligations/guidelines, in addition to the provisions in the “Intellectual Property Option to Collaborator” terms of award modifications, apply to the use of BAY 43-9006 in this study:

1. BAY 43-9006 may not be used for any purpose outside the scope of this protocol, nor can BAY 43-9006 be transferred or licensed to any party not participating in the clinical study. Collaborator data for BAY 43-9006 are confidential and proprietary to Collaborator and shall be maintained as such by the investigators.

2. For a clinical protocol where there is an investigational agent used in combination with (an)other investigational agent(s), each the subject of different CTAs, the access to and use of data by each Collaborator shall be as follows (data pertaining to such combination use shall hereinafter be referred to as "Multi-Party Data”.):

a. NCI must provide all Collaborators with prior written notice regarding the existence and nature of any agreements governing their collaboration with NIH, the design of the proposed combination protocol, and the existence of any obligations which would tend to restrict NCI's participation in the proposed combination protocol.

b. Each Collaborator shall agree to permit use of the Multi-Party Data from the clinical trial by any other Collaborator solely to the extent necessary to allow said other Collaborator to develop, obtain regulatory approval, or commercialize its own investigational agent.

c. Any Collaborator having the right to use the Multi-Party Data from these trials must agree in writing prior to the commencement of the trials that it will use the Multi-Party Data solely for development, regulatory approval, and commercialization of its own investigational agent.

3. Clinical Trial Data and Results and Raw Data developed under a CTA will be made available exclusively to Collaborator, the NCI, and the FDA, as appropriate.

4. When a Collaborator wishes to initiate a data request, the request should first be sent to the NCI, who will then notify the appropriate investigators (Group Chair for Cooperative Group studies, or PI for other studies) of Collaborator's wish to contact them.

5. Any data provided to Collaborator for Phase 3 studies must be in accordance with the guidelines and policies of the responsible Data Monitoring Committee (DMC), if there is a DMC for this clinical trial.

1. Any manuscripts reporting the results of this clinical trial should be provided to CTEP for immediate delivery to Collaborator for advisory review and comment prior to submission for publication. Collaborator will have 30 days from the date of receipt for review. Collaborator shall have the right to request that publication be delayed for up to an additional 30 days in order to ensure that Collaborator’s confidential and proprietary data, in addition to Collaborator’s intellectual property rights, are protected. Copies of abstracts should be provided to CTEP for forwarding to Collaborator for courtesy review as soon as possible and preferably at least three (3) days prior to submission, but in any case, prior to presentation at the meeting or publication in the proceedings. Copies of any manuscript and/or abstract should be sent to:

Regulatory Affairs Branch, CTEP, DCTD, NCI

Executive Plaza North, Room 718

Bethesda, Maryland 20892

FAX 301-402-1584

The Regulatory Affairs Branch will then distribute them to Collaborator. No publication, manuscript or other form of public disclosure shall contain any of Collaborator’s confidential/ proprietary information.

**11. STATISTICAL CONSIDERATIONS**

**11.1 Endpoints**

Primary endpoint:

- Response rate (RR)

Secondary endpoints:

- Time to progression (TTP)
- Toxicity
- Correlative Data
  1. **Study Design / Stratification Factors**

*Clinical Endpoints*

The primary endpoint of this trial is response rate defined as is either a complete or a partial response at the end of second cycle, using the RECIST criteria.

The response rate for standard of care DTIC is 5-10%. If the response is < 5%, the new treatment will not be considered for further study.

In each subgroup defined by BRAF status, a minimax two-stage Simon design will be used to test the null hypothesis that response rate at the end of second cycle (56 days) is p ≤ 0.05 versus the alternative that the response rate is p ≥ 0.20. If the drug is actually not effective, there is 3.3% probability of concluding that it is (the target was 5%). If the drug is actually effective there is a 14.2% probability of concluding that it is not (the target was 20%). After testing the drug on 13 patients per BRAF subgroup in the first stage, the trial will be terminated if 0 patients per subgroup respond. If the trial goes on to the second stage, a total of 37 patients per BRAF subgroup will be studied. If the total number responding is less than or equal to 4 patients per subgroup, the drug is rejected for that subgroup.

The overall response rate along with subgroup-specific response rates will be estimated at the end of the trial along with 95% confidence interval.

*Correlative Study Endpoints*

An important goal of this trial is to evaluate the biological markers pre and post treatment. Eligibility for the first stage of the trial (first 13 patients in each subgroup) requires that patients have biopsiable sites of disease. Tumor tissue will be collected and assessed for change in BRAF, P-MAPK, CDK4, and Cyclin D1 levels pre versus post treatment by analyzing the changes in the intensity of the specific bands on immunoblot. This is usually done visually (increase, no change, decrease) but can be quantified using a digital scanner. It is critically important to ensure that equal amounts of protein are loaded on to each lane. This can be confirmed by comparing the intensity of the p85 PI3k bands. The proportion of patients with decreases in levels of BRAF, CDK4, or phospho-MAPK will be estimated along with 95% confidence intervals.

Cyclin D1 and phospho-MAPK expression will also be assessed by immunohistochemistry. The percentage of tumor cells staining for either marker will be quantified visually and expressed as a percentage of positively-staining tumor cells. In this study, we are measuring phospho-MAPK both by immunoblotting and by immunohistochemistry. These two techniques are complimentary in that immunoblotting measures the relative amount of phospho-MAPK in the tumor while immunohistochemistry gives information as to the percentage of cells expressing the protein.

**11.3 Sample Size/Accrual Rate**

A total sample size of 37 eligible patients per subgroup is planned. Accrual of 4-5 patients per month is expected, so that the study should be completed in, at most, 24 months.

**11.4 Analysis of Secondary Endpoints**

All patients will be followed for progression and survival. Kaplan-Meier estimates will be calculated for time to progression and overall survival, and medians, along with two-sided 95% confidence intervals, will be reported.

All adverse events without regard to causal relationship and by causal relationship to study drugs will be summarized.

Exploratory analyses of correlative endpoints will be examined over time using descriptive statistics and graphical displays. In particular, these endpoints will also be explored in relation to response to treatment and tumor burden.

**11.5 Reporting and Exclusions**

**11.5.1 Toxicity**

All patients will be evaluable for toxicity from the time of their first treatment with BAY.

- - 1. **Evaluation of response**

All patients included in the study must be assessed for response to treatment, even if there are major protocol treatment deviations or if they are ineligible. Each patient will be assigned one of the following categories: 1) complete response, 2) partial response, 3) stable disease, 4) progressive disease, 5) early death from malignant disease, 6) early death from toxicity, 7) early death because of other cause, or 9) unknown (not assessable, insufficient data). [Note: By arbitrary convention, category 9 usually designates the “unknown” status of any type of data in a clinical database.]

All of the patients who met the eligibility criteria should be included in the main analysis of the response rate. Patients in response categories 4-9 should be considered as failing to respond to treatment (disease progression). Thus, an incorrect treatment schedule or drug administration does not result in exclusion from the analysis of the response rate. Precise definitions for categories 4-9 will be protocol specific.

All conclusions should be based on all eligible patients. Descriptive analyses will also be provided for patient subsets that exclude patients for whom major protocol deviations have been identified (e.g., early death due to other reasons, early discontinuation of treatment, major protocol violations, etc.). The reasons for excluding patients from the analysis should be clearly reported.

**12. REFERENCES**

1.Roth A and Kirkwood J: Melanoma: Classic approaches to management and current integration of biologic response modifiers. Current Concepts Oncol. 9:1-11. 1988.

2.Houghton AN, Legha S, Bajorin DF: Chemotherapy for metastatic melanoma, in *Cutaneous Melanoma*, Balch C, Houghton A, Milton, Sober, Soong (eds). 2^nd^ Edition, J.B. Lippincott, Philadelphia, pp. 498-508, 1992.

3.Middleton MR, Gore M, Tilgen W, et al.: Randomized, phase III study of temozolomide (TMZ) versus dacarbazine (DTIC) in the treatment of patients with advanced, metastatic melanoma. J Clin Oncol. 18:158-166, 2000. See also Cohen MH and Johnson JR: Temozolomide for advanced metastatic melanoma (letter), J Clin Oncol. 18:2185, 2000.

4.Legha SS, Ring S, Papadopoulos N, Plager C. Chawla S, Benjamin R: A prospective evaluation of a triple‑drug regimen containing cisplatin, vinblastine and DTIC (CVD) for metastatic melanoma. Cancer 64:2024, 1989.

5.DelPrete SA, Maurer LH, O'Donnell J: Combination chemotherapy with cisplatin, carmustine, dacarbazine and tamoxifen in metastatic melanoma. Cancer Treat Rep. 69:1403. 1984.

6.Buzaid AC, Legha S, Winn R, Belt R, et al.: Cisplatin (C),vinblastine (V), and dacarbazine (D) (CVD) versus dacarbazine alone in metastatic melanoma: Preliminary results of a phase III cancer community oncology program (CCOP) trial. Proc Am Soc Clin Oncol. 12:389, 1993.

7.Cocconi G, Bella M, Calabresi F, et al.: Treatment of metastatic malignant melanoma with dacarbazine plus tamoxifen. N Engl J Med. 327:516-523, 1992.

8.Falkson, CI, Ibrahim, J, Kirkwood J, Blum, R: A randomized phase III trial of dacarbazine (DTIC) versus DTIC + interferon alpha-2b (IFN) versus DTIC + tamoxifen (TMX) versus DTIC + IFN + TMX in metastatic malignant melanoma: an ECOG trial. Proc Am Soc Clin Oncol. 15:435, 1996.

9.McClay E, Mastrangelo M, Bellet R, et al.: An effective chemo/hormonal therapy regimen for the treatment of disseminated malignant melanoma. Proc Am Soc Clin Oncol. 8:282, 1989.

10.Kirkwood JM: Studies of interferon in the therapy of melanoma. Semin Oncol. (suppl 7) 18:83-89, 1991.

11.Sparano JA and Dutcher JP: Interleukin-2 for the treatment of advanced melanoma. Hematol Oncol. Annals 1:279-285, 1993.

12.Dutcher JP, Creekmore S, Weiss GR, et al.: Phase II study of high-dose interleukin-2 and lymphokine activated killer cells in patients with metastatic malignant melanoma. J Clin Oncol. 7:477-485, 1989.

13.Parkinson DR, Abrams JS, Wiernik PH, et al.: Interleukin-2 therapy in patients with metastatic malignant melanoma: a phase II study. J Clin Oncol. 8:1650‑1656, 1990.

14.Rosenberg SA, Yang JC, Topalian SL: Treatment of 283 consecutive patients with metastatic melanoma or renal cell cancer using high-dose bolus interleukin-2. JAMA 907-913, 1994.

15.Campbell, S.L., R. Khosravi-Far, K.L. Rossman*, et al.* (1998). Increasing complexity of Ras signaling.  *Oncogene*. 17:1395-1413.

16.Chong, H., H.G. Vikis, and K.L. Guan. (2003). Mechanisms of regulating the Raf kinase family.  *Cell Signal*. 15:463-469.

17.Davies, H., G.R. Bignell, C. Cox*, et al.* (2002). Mutations of the BRAF gene in human cancer.  *Nature*. 417:949-954.

18.Mercer, K.E., and C.A. Pritchard. (2003). Raf proteins and cancer: B-Raf is identified as a mutational target.  *Biochim Biophys Acta*. 1653:25-40.

19.Pollock, P.M., and P.S. Meltzer. (2002). A genome-based strategy uncovers frequent BRAF mutations in melanoma. *Cancer Cell*. 2:5-7.

20.Singer, G., R. Oldt, 3rd, Y. Cohen*, et al.* (2003). Mutations in BRAF and KRAS characterize the development of low-grade ovarian serous carcinoma. *J Natl Cancer Inst*. 95:484-486.

21.Tannapfel, A., F. Sommerer, M. Benicke*, et al.* (2003). Mutations of the BRAF gene in cholangiocarcinoma but not in hepatocellular carcinoma.  *Gut*. 52:706-712.

22.Cohen, Y., M. Xing, E. Mambo*, et al.* (2003). BRAF mutation in papillary thyroid carcinoma.  *J Natl Cancer Inst*. 95:625-627.

23.Strumberg, D., A. Awada, M. Piccart, *et al.* (2003). Final report of the phase I clinical program of the novel Raf kinase inhibitor BAY 43-9006 in patients with refractory solid tumors. *Proc Amer Soc Clin Oncl*. 22:A813.

24. Wong, K-K., J.P. Eder, J. Clark, *et al*. (2003). Final results of a phase I study to determine the safety, maximum tolerated dose, pharmacokinetics and pharmacodynamics of BAY 43-9006 in repeated cycles of 1 week/1 week off in patients with advanced, refractory solid tumors. *Proc Amer Soc Clin Oncl*. 22:A976.

25.Siu, L., A. Awada, C.H. Takimoto, *et al*. (2003). Phase I study of oral raf-1 kinase inhibitor BAY 43-9006 with gemcitabine in patients with advanced solid tumors. *Proc Amer Soc Clin Oncl*. 22:A828.

26.Flaherty, K.T., R.J. Lee, R. Humphries, *et al.* (2003). Phase I trial of BAY 43-9006 in combination with carboplatin (C) and paclitaxel (P). *Proc Amer Soc Clin Oncl*. 22:A2854.

27.Drobnjak M, Osman I, Scher HI, Fazzari F, Cordon-Cardo C. Overexpression of cyclin D1 is associated with metastatic prostate cancer to bone. Clin Cancer Res 2000; 6:1891-5.

28.Kim SH, Lewis JJ, Brennan MF, Woodruff JM, Dudas M Cordon-Cardo C. Overexpression of cyclin D1 is associated with poor prognosis in extremity soft tissue sarcomas. Clin Cancer Res 1998; 4:2377-82.

29.Osman I, Scher HI, Zhang F, et al. Expression fo cyclin D1, but not cyclins E and A, is related to progression in bilharzial bladder cancer. Clin Cancer Res 1997; 3: 2247-51.

30. Gordon, et. al. Cancer Res 2003 Jul 15; 63(14):3955-7.

31.Schittny JC and Yurchenco, P. Basement membranes: molecular organization and function in development and disease. Curr. Opin. Cell Biol., *1:* 983-988, 1989.

32.Adams, J. and Watt, F. Regulation of development and differentiation by the extracellular matrix. Development, *117:* 1183-1198, 1993.

33.Sternlicht, M. and Werb, Z. How matrix metalloproteinases regulate cell behavior. Ann. Rev. Cell Dev. Biol., *17:* 463-516, 2001.

34.Xu JD, Rodriguez E, Petitclere J, Kim J, Hangai S, Yuen S, Davis G., and Brooks, P. Proteolytic exposure of a cryptic site within collagen type IV is required for angiogenesis and tumor growth in vivo. J. Cell Biol., *154:* 1069-1079, 2001.

35.Kymala T, Tammela TL, Risteli L, Risteli J, Kontturi M, and Elomala I. Type-I collagen degradation product (1CTP) gives information about the nature of bone metastasis and has prognostic value in prostate cancer. Brit. J. Cancer, *71:* 1061-1070, 1995.

36.Nakashima J, Sumitomo M, Miyajima A, Jitsukawa, S., Saito S, Tachibana M, and Murai M. The value of serum carboxyterminal propeptide of type-I procollagen in predicting bone metastases in prostate cancer. J. Urol., *157:* 1736-1739, 1997.

# APPENDIX A

**ELIGIBILITY CRITERIA**

| Confirmed Stage IV Melanoma |  |
| --- | --- |
| Measurable Disease |  |
| No prior therapy |  |
| Tumor amenable to biopsy |  |
| Scans within 4 weeks |  |
| ECOG Performance Status 0-2 |  |
| ANC > 1.5 x 10^9^/l |  |
| Platelets > 100 x 10^9^/l |  |
| Total Bilirubin < 2 x ULN |  |
| AST/ALT < 2.5 x ULN |  |
| Creatinine < 1.5 x ULN |  |
| No Brain Mets or Stable Brain Mets for at least 6 weeks after whole brain radiation |  |
| Not pregnant |  |
| No uncontrolled medical illnesses |  |
| Signed informed consent |  |

**Appendix B**

**Performance Status Criteria**

| **ECOG Performance Status Scale** | | **Karnofsky Performance Scale** | |
| --- | --- | --- | --- |
| Grade | Descriptions | Percent | Description |
| 0 | Normal activity. Fully active, able to carry on all pre-disease performance without restriction. | 100 | Normal, no complaints, no evidence of disease. |
|  |  | 90 | Able to carry on normal activity; minor signs or symptoms of disease. |
| 1 | Symptoms, but ambulatory. Restricted in physically strenuous activity, but ambulatory and able to carry out work of a light or sedentary nature (e.g., light housework, office work). | 80 | Normal activity with effort; some signs or symptoms of disease. |
|  |  | 70 | Cares for self, unable to carry on normal activity or to do active work. |
| 2 | In bed <50% of the time. Ambulatory and capable of all self-care, but unable to carry out any work activities. Up and about more than 50% of waking hours. | 60 | Requires occasional assistance, but is able to care for most of his/her needs. |
|  |  | 50 | Requires considerable assistance and frequent medical care. |
| 3 | In bed >50% of the time. Capable of only limited self-care, confined to bed or chair more than 50% of waking hours. | 40 | Disabled, requires special care and assistance. |
|  |  | 30 | Severely disabled, hospitalization indicated. Death not imminent. |
| 4 | 100% bedridden. Completely disabled. Cannot carry on any self-care. Totally confined to bed or chair. | 20 | Very sick, hospitalization indicated. Death not imminent. |
|  |  | 10 | Moribund, fatal processes progressing rapidly. |
| 5 | Dead. | 0 | Dead. |

**Appendix C**

**Summary of Studies in Support of Genetic and Pharmacodynamic Endpoints:**


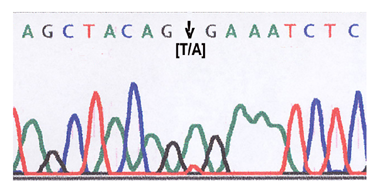


**Figure 1. BRAF HotSpot Mutation.** An electropherogram demonstrating the T1796A nucleotide substitution detected in a metastatic melanoma tissue sample.

The laboratories of Isman Osman and David Polsky at NYU Medical center have recently reported on a series of analyses of *BRAF* and *N-RAS* mutations in 77 metastatic melanoma tumor specimens, and 11 melanoma cell lines^1^ (Cancer Res 63: 3955-3957, 2003, see Appendix II). In this study they amplified and sequenced *BRAF* exons 11 and 15, and *N-RAS* exons 2 and 3. They were able to detect mutations in 36 of 77 (47%) tissue specimens and 8 of 11 (73%) cell lines. Ninety-two percent of mutations in tissues occurred in *BRAF* exon 15, and 31 of 33 (94%) while exon 15 mutations consisted of the previously identified T1796A nucleotide substitution. An example of this mutation is shown in Figure 1.

In other preliminary studies, Drs. Osman and Polsky have developed a PCR amplification strategy that specifically amplifies the T1796A mutant. They have designed three different oligonucleotide primers specific for the mutation (mFwd, mRev1, mRev2). These mutant specific primers are used with intronic primers to amplify products that range in size from 151 bp to 172 bp. Our colleagues had established the specificity of all three of the mutant-specific primers by testing them on melanoma cell lines SK-MEL 85 (wild-type for exon 15, mutant for exon 11), and SK-MEL 29 (mutant for exon 15). As shown in Figure 2, PCR reactions using each primer set were performed at annealing temperatures of 58^o^C, 60^o^C, and 63^o^C. Each primer showed specificity for the mutant product, as evidenced by disappearance of the wild-type band as the temperature increased (compare lanes 1, 4, and 7 (mutant) with lanes 2, 5 and 8 (wild-type). As part of the clinical study validation protocol the assay will be optimized for the assessments in tumor tissues along with peripheral blood nuclear cells.

58^o^C60^o^C 63^o^C

12345678 9M

400bp

200bp

100bp

261bp

168bp

261bp

172bp

**Figure 2. Multiplex PCR for mutant BRAF and p53 exon 4 (internal control).** DNA from SK-Mel 29 (BRAF mutant), lanes 2, 5, 8 or SK-Mel 85 (BRAF wild-type), lanes 3, 6, 9 was used as template to test the three mutant specific primers at 3 different annealing temperatures (Ta). Lanes 1-3, Ta=58^o^C; 4-6, Ta=60^o^C; 7-9, Ta=63^o^C. Each panel shows the result from a different primer. A. mRev2, B. mRev1, C. mFwd. In all three panels, the lower band is the product of the BRAF primers, the upper band, the p53 primers. Lanes 1, 4, and 7 are negative controls in which only the template DNA was omitted from the reaction.


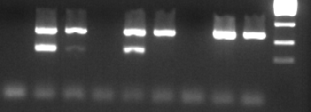

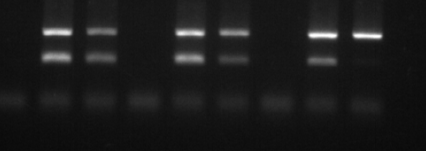

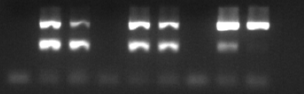


**A**

**B**

**C**

Additional confirmation of the specificity of these primers will be obtained by analyzing a series of 9 other melanoma cell lines available in Dr. Polsky’s laboratory. In addition, the utility of the assay will be extended further with the use of fluorescently labeled intronic primers that should enhance the sensitivity and specificity. Our collaborators estimate that fluorescent detection of PCR products will be approximately 200-fold more sensitive than detection using ethidium bromide-stained agarose gels. An example of this point is demonstrated in Figure 3 where serial dilutions of a fluorescently labeled PCR product were analyzed in an ethidium bromide-stained agarose gel and compared with the ABI 310 Genetic analyzer. Quantitation of the PCR products in the agarose gel using a molecular mass ladder showed that reliable detection of the PCR products was far superior using the ABI 310 analyzer compared to the agarose gel.


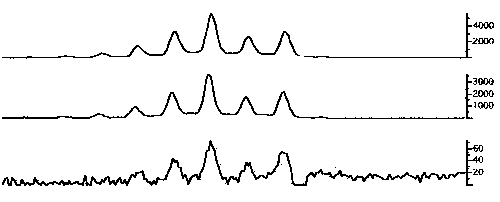

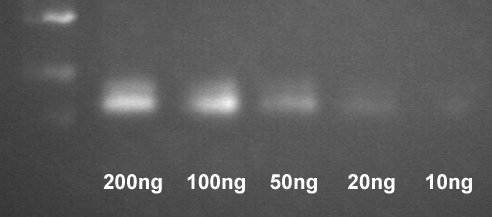


20ng

10ng

0.1ng

Figure 3. Sensitivity detection of PCR products using ethidium bromide stained agarose gels vs. an ABI 310 Genetic Analyzer. **Dilutions of fluorescently tagged PCR products from a tumor sample heterozygous for the microsatellite marker D9S2136 were analyzed using a 1.5% agarose gel (top panel) and ABI 310 Genetic Analyzer (bottom panel). Reliable detection in agarose is limited to amounts of 20ng or greater, whereas the sample shows peaks at 3 times baseline at 0.1ng, a 200-fold improvement in detection.**

**Mass**

**Ladder**

134bp 138 bp

**Collection of samples**

# Tumor samples

Pre-treatment samples can be collected up to 2 weeks prior to starting treatment. The post-treatment sample should be collected 18-48 hours after the first treatment cycle. Although in some patients it will be possible to sample the same tumor pre-treatment and post-treatment, in most patients different tumors will be sampled. It is ideal to obtain multiple pre-treatment and post-treatment samples, an attempts will be made to carry this out.

The pre-treatment and post-treatment melanoma tumor samples will fixed in formaline and parffim embedded. Samples will be trimmed of obvious non-melanoma tissue. If there is a sufficient amount of normal skin associated with the sample, the normal skin may be stored separately and analyzed. Ideally, tumors biopsy will be approximately 1cm3. If larger samples are obtained, they will be split in duplicate. Blocks will be labeled with the patient’s name, protocol site (e.g NYU, Chicago university, Cornell ), date of collection, and an indication whether the sample was pretreatment or post-treatment.

**PBMC**

One tube of blood will be collected using Vacutainer CPT tubes (Becton Dickinson, Franklin Lakes, NJ) at the time of tumor collection, and just prior to treatment #2. In patients not undergoing tumor biopsy, PBMC will be collected just prior to treatment #1, 18-48 hours later, and just prior to treatment #2. The cells are processed as detailed in the Appendix describing the CPT processing steps.

**Appendix D**

**Tumor Immunohistochemistry**

Immunohistochemistry of Cyclin D1: Staining for cyclin D1 will be carried out in Dr. Osman’s laboratory.  Formalin-fixed paraffin-embedded sections will be cut at 4-5 microns placed on Superfrost/Plus slides (Fisher) and baked at 60^o^C for at least 60 minutes. Slides will be deparaffinized and hydrated in distilled water. Then the slides will be washed, quenched of endogenous peroxidase, and washed again in saline followed by phosphate-buffered saline (PBS). Slides will be then placed in a solution containing 0.005% bovine serum albumin (BSA) followed by normal serum at 1:20 dilution in 2% BSA/PBS. A 150 mLvolume of a 1:100 dilution of the primary antibody is placed on the tissue and incubated overnight (15-18 hours) at 40^o^C in a humidity chamber. After several washes in PBS, a secondary antibody is applied for 60 minutes at a dilution of 1:500 in 1% BSA/PBS.  Peroxidase-conjugated streptavidin (1:500 in 1% BSA/PBS) is then applied for 45-60 minutes, followed by several washes in PBS. Diaminobenzidine at 0.06% is used as the chromogen, and Harris-modified hematoxylin (Fisher) is used for counterstain. In addition, we will measure phosphorylated-MAPK and phosph-ERK by immunohistochemistry using an antibody to the activated, phosphorylated MAPK (Cell Signaling Technology, Beverly, MA). For positive Cyclin D1 controls, we typically use a positive bladder cancer or prostate cancer specimen, as we have described previously(27,29).  Prostate cancer is typically positive for phospho-ERK staining and we plan to identify tumor samples that will serve as positive controls.  Negative controls are specimens stained with isotype-specific control primary antibody. We will obtain a quantitative assessment of the degree of antibody staining employing Kodak ID image analysis software.

Appropriate antigen retrieval will be performed for each of the markers in 0.1mM citrate buffer at pH 6. The immunostaining will be performed using the avidin biotin peroxidase complex method (ABC) using a semi-automated immunostainer (NexES, Ventana Medical Systems, Tucson, AZ). Secondary detection uses conventional, well-established avidin-biotin horseradish peroxidase complex with 3,3 diaminobenzidine (DAB) as the chromogen. The immunohistochemistry images from five defined regions of the stained tumor slides are to be captured at 60X magnification using an Olympus BX40 microscope and DP11 digital image system as JPEG files.

The intensity of immunohistochemical staining for each of the respective markers is quantified via a three step procedure that involves the following: 1) The analysis of the JPEG photomicrograph image using Adobe Photoshop (San Jose, Ca) to process the immunohistochemictry densities through the use of the magic wand tool to extract the stained tissue color essentially as detailed by Matlowsky et al (2004) followed saving the processed image in a TIFF file format. 2) The Photoshop processed images were analyzed using Kodak ID image analysis software (Kodak Imaging systems, Rochester, NY) employing the “density slice method” for quantification of the pixel density in the regions of interest (ROI) correlating to the immunohistochemistry positive staining for each marker. 3) The “summation of intensity values” from the Kodak ID density table were exported via a text format to Microsoft Excel (Microsoft Corp, Redman Washington) where the summation of the identified ROIs was carried out.

Matkowsky K, Glover S, Benya R: Quantitative Immunohistochemistry: A New Algorithm Measuring Cumulative Signal Strength and Receptor Number. Microscopy and Analysis 18:5-6, 2004

**Appendix E**

**Preparation of mononuclear cells for DNA extraction and plasma separation and storage for later follow-up studies**

1. After collection, gently invert each tube 4 to 5 times to mix anticoagulant additive with blood.
2. Keep the tubes upright at room temperature until centrifugation. **Centrifuge blood samples within 1 hour of collection.**
3. Immediately before centrifugation, remix the blood sample by gently inverting the tubes 4 to 5 times.
4. Centrifuge in a temperature controlled (refrigerated) horizontal rotor, (swing-out head) centrifuge (18-20^o^C)for 20 minutes at 1700 x g .
5. After centrifugation, resuspend the cells into the plasma by inverting the unopened “green/red-top”CPT tubes gently 4 times remove stopper and transfer into a 15 ml polypropylene tube.
6. Centrifuge the tube containing the PBMCs and plasma at 1000 x g for 5 minutes.
7. Remove the supernatent containing the plasma and transfer to a 5 ml freezer vial.
8. Add 10 ml of PBS to the tube still containing the mononuclear pellet and resuspend with gentle vortex mixing.
9. Centrifuge the PBS tube containing the PBMCs at 1000 x g for 5 minutes. Repeat steps 8 and 9 one more time.
10. Resuspend the mononuclear cells in 1 ml of PBS with gentle vortexing.
11. Transfer the mononuclear cell suspension to a 1.5 ml Epindorf tube and store at –20^o^C.

**APPENDIX F**

**Monitoring of Collagen Cryptic Epitopes in Serum and Urine:**

Standard ELISA methods have been used in the development of the cryptic collagen ELISA Assay. Briefly, Mabs HUI77 or HUIV26 are immobilized on 96-well microtiter plates. The plates are next washed with buffer and incubated with 5% BSA in PBS for 1.5 hours to block non-specific binding sites. Serum samples are diluted in 1:2 in dilution buffer and 50µl is added to each well and allowed to incubate for 2 hours at room temperature. The plates are next washed 8 times with wash buffer and incubated with a biotinylated polyclonal antibody to collagen for 2 hours. The plates are washed again and incubated with HRP-conjugated anti-Biotin antibody for 2 hours. Finally, the plates are washed and peroxidase substrate is added. The optical density is measured at a wavelength of 492 nm. Serum and urine will be collected for monitoring at day 6 of each cycle following treatment. The amount of denatured type IV collagen in ng/ml will be assessed.

**APPENDIX G**

**SAMPLE DATA COLLECTION FORM**

NYU 04- Guidelines for Data Collection

Patient Registration:

After patient has been screened for eligibility and signed an informed consent, Patient Registration and Eligibility Checklist forms will be sent via fax or email to the Coordinating Center. The Coordinating Center will return the Patient Registration form with the assigned patient id number.

A copy of the informed consent together with copies of source documents confirming eligibility will be sent via fax or mail as soon as possible after the patient is on-study.

Data Collection:

An EXCEL spreadsheet template will be sent electronically to each site. The information collected on the spreadsheet is entered into the NCI CDUS web-based application and submitted to the NCI quarterly on the following schedule:

Submission Deadline Period Covered

30 April 01 Jan - 31 Mar

31 July 01 Mar - 30 Jun

31 October 01 Jul - 30 Sep

31 January 01 Oct - 31 Dec

Updated spreadsheets will be sent to the Coordinating Center on a regular basis and at least 2 weeks before each NCI submission deadline.

In addition to the spreadsheet, copies of source documents will be sent via fax or mail to the Coordinating Center when a patient goes off-study or more frequently for responders.

# ON-STUDY REGISTRATION

| NYU 04- A Phase II Study of BAY 43-9007 in Stage IV Malignant Melanoma | | | |
| --- | --- | --- | --- |
| Date Submitted:  Mo / Dy / Yr ___/ ___/ ___ | NCI Protocol #: | Patient Initials: | |
| SEX:  [ ] M [ ] F | Date of Birth:  Day/ Mon / Yr  ___/ ____/ ___ | Age (yrs) | Ethnicity: |
| Body Weight (Kg) | | Height (cm) | |
| Method of Payment: | | Postal Code: | |
| Histology:  **Melanoma** | | Performance Status: | |
| Date of Diagnosis:  Mo / Dy / Yr: _____/ _____/ _____ | | Date On-Study:  Mo / Dy / Yr: _____/ _____/ _____ | |
| Prior Therapy  Chemo [ ] Y [ ] N Hormone [ ] Y [ ] N Immuno [ ] Y [ ] N  Radiation [ ] Y [ ] N Surgery [ ] Y [ ] N | | | |

Patient ID Number: _________________ (assigned at Coordinating Center)

Completed By:

Name: ____________________ Signature: ___________________ Date: ___/ ___/ ___

**Appendix H**

**Medication Diary**

| **Name** |  |  |  |  |  |  |
| --- | --- | --- | --- | --- | --- | --- |
| **1**  **AM_______**  **PM_______** | **2 AM_______**  **PM_______** | **3 AM_______**  **PM_______** | **4 AM_______**  **PM_______** | **5 AM_______**  **PM_______** | **6 AM_______**  **PM_______** | **7 AM_______**  **PM_______** |
| **8 AM_______**  **PM_______** | **9 AM_______**  **PM_______** | **10 AM_______**  **PM_______** | **11 AM_______**  **PM_______** | **12 AM_______**  **PM_______** | **13 AM_______**  **PM_______** | **14 AM_______**  **PM_______** |
| **15 AM_______**  **PM_______** | **16 AM_______**  **PM_______** | **17 AM_______**  **PM_______** | **18 AM_______**  **PM_______** | **19 AM_______**  **PM_______** | **20 AM_______**  **PM_______** | **21 AM_______**  **PM_______** |
| **22 AM_______**  **PM_______** | **23 AM_______**  **PM_______** | **24 AM_______**  **PM_______** | **25 AM_______**  **PM_______** | **26 AM_______**  **PM_______** | **27 AM_______**  **PM_______** | **28 AM_______**  **PM_______** |
